# Long-Term Glycemic Control Improvement After the Home and Self-Care Program for Patients With Type 1 Diabetes: Real-World–Based Cohort Study

**DOI:** 10.2196/60023

**Published:** 2024-09-11

**Authors:** Dae-Jeong Koo, Sun-Joon Moon, Suhyeon Moon, Se Eun Park, Eun-Jung Rhee, Won-Young Lee, Cheol-Young Park

**Affiliations:** 1 Division of Endocrinology and Metabolism Department of Internal Medicine Changwon Fatima Hospital Changwon Republic of Korea; 2 Division of Endocrinology and Metabolism Department of Internal Medicine, Kangbuk Samsung Hospital Sungkyunkwan University School of Medicine Seoul Republic of Korea; 3 Division of Biostatistics Department of Academic Research Kangbuk Samsung Hospital Seoul Republic of Korea

**Keywords:** type 1 diabetes, structured education, home health care, glycated hemoglobin, continuous glucose monitoring, mobile phone

## Abstract

**Background:**

The prevalence of type 1 diabetes (T1D) is increasing worldwide, with a much higher proportion of adult patients. However, achieving stable glycemic control is difficult in these patients.

**Objective:**

After periodic implementation of structured education for patients with T1D through the Home and Self-Care Program, a pilot home health care project promoted by the Korean government, we evaluated the program’s effects on glycemic control.

**Methods:**

This study was conducted from April 2020 to March 2023. We analyzed 119 participants with T1D aged >15 years. Nursing and nutrition education were provided separately up to 4 times per year, with physician consultation up to 6 times per year. A distinguishing feature of this study compared with previous ones was the provision of remote support using a general-purpose smartphone communication app offered up to 12 times annually on an as-needed basis to enhance the continuity of in-person education effects. Patients were followed up on at average intervals of 3 months for up to 24 months. The primary end point was the mean difference in glycated hemoglobin (HbA_1c_) at each follow-up visit from baseline. For continuous glucose monitoring (CGM) users, CGM metrics were also evaluated.

**Results:**

The mean HbA_1c_ level of study participants was 8.6% at baseline (mean duration of T1D 10.02, SD 16.10 y). The HbA_1c_ level reduction in participants who received at least 1 structured educational session went from 1.63% (SD 2.03%; *P*<.001; adjustment model=1.69%, 95% CI 1.24%-2.13% at the first follow-up visit) to 1.23% (SD 1.31%; *P*=.01; adjustment model=1.28%, 95% CI 0.78%-1.79% at the eighth follow-up visit). In the adjustment model, the actual mean HbA_1c_ values were maintained between a minimum of 7.33% (95% CI 7.20%-7.46% at the first follow-up visit) and a maximum of 7.62% (95% CI 7.41%-7.82% at the sixth follow-up visit). Among CGM users, after at least 1 session, the mean time in the target range was maintained between 61.59% (adjusted model, 95% CI 58.14%-65.03% at the second follow-up visit) and 54.7% (95% CI 50.92%-58.48% at the eighth follow-up visit), consistently staying above 54.7% (corresponding to an HbA_1c_ level of <7.6%). The mean time below the target range (TBR) also gradually improved to the recommended range (≤4% for TBR of <70 mg/dL and ≤1% for TBR of <54 mg/dL).

**Conclusions:**

The Home and Self-Care Program protocol for glycemic control in patients with T1D is effective, producing significant improvement immediately and long-term maintenance effects, including on CGM indexes.

## Introduction

### Background

As of 2021, approximately 8.4 million people worldwide are living with type 1 diabetes (T1D). Among them, only approximately 18% are aged <20 years [1]. Because the incidence of T1D is increasing worldwide, there is a need for comprehensive, tailored interventions that consider various needs for disease management, along with policy support from governments and the establishment of clinical evidence to support these interventions [2].

Research from the T1D Exchange Registry shows that only 17% of children and 21% of adults currently succeed in reducing their glucose to target levels [3]. Despite such a low glycemic control rate, level-2 hypoglycemia (<54 mg/dL) occurs quite frequently, ranging from once every 6 days to once every 2 days, in patients receiving multiple daily insulin injection treatment [4,5]. Due to the complexity and difficulty of managing T1D, it is well established that proper structured education is very important for controlling glucose levels and improving quality of life (QOL) [6-9]. A variety of programs, including education and support programs, technological interventions, psychosocial interventions, and transitional care programs, have been developed and applied to improve glycemic control or QOL in young adult patients with T1D [10]. Those interventions can be effective, but their effectiveness can vary depending on the specific design of the intervention and how it is implemented [10]. Many studies show promise, but further research is needed to identify the most effective strategies. The need for longitudinal studies to assess the long-term effects of these interventions is particularly great, along with studies based on personalized and integrated interventions that use a multifaceted approach [10]. However, clinic hours and platforms for structured education, which are needed to provide individualized and comprehensive treatment for patients with T1D, are lacking.

### Objectives

Therefore, the Korean government promoted a pilot home health care project for patients with T1D in January 2020. The purpose of this project was to develop a program that continuously manages patients and provides feedback even when they are at home, thereby reducing unnecessary medical costs due to worsening disease and simultaneously improving the QOL of patients. Specifically, the goal was to develop periodic medical interventions that enable home health care services, including education and counseling for self-management and regular monitoring of conditions. Our team at Kangbuk Samsung Hospital participated in this project and developed a comprehensive and structured education program to meet those needs (tentative name *Home and Self-Care Program* [HELP]) that includes remote support using an existing universal smartphone app. For this paper, we evaluated the short- and long-term effects of HELP on glycemic control in patients with T1D from adolescence to middle age.

## Methods

### Study Setting and Context

Kangbuk Samsung Hospital is a prominent health care institution located in Seoul, South Korea. It is affiliated with Sungkyunkwan University and serves as a tertiary teaching hospital. The hospital is equipped with 700 beds and caters to a wide range of medical needs, providing advanced health care services to its patients. The hospital mainly serves patients referred from primary or secondary hospitals, ensuring that they receive specialized care. Before the implementation of HELP, the team included 5 diabetologists among 7 endocrinologists, 2 education nurses, and 1 nutritionist. During the HELP period, the team was expanded to include 3 education nurses and 2 nutritionists.

Kangbuk Samsung Hospital had limited structured education programs for patients with T1D before the implementation of HELP. Several factors contributed to this limitation. The standard practice in South Korea of having approximately 5-minute clinic visits per patient due to low fees for service and the need to see many patients resulted in insufficient time to provide comprehensive education and support during appointments. In addition, there were scarce structured education programs available for patients with T1D, which hindered the ability to deliver consistent and thorough education on diabetes management. The lack of a fee-for-service model or specific education fees for T1D care made it challenging to allocate resources and time for comprehensive patient education. In the clinic, insulin dosage adjustments were provided by physicians primarily based on laboratory test results and daily log data from patients. Specific education for T1D was scarce at the time.

Despite these challenges, our hospital’s team of endocrinologists, education nurses, and nutritionists strived to manage patients with T1D to the best of their abilities within these constraints. The introduction of the HELP program aimed to address these gaps and provide a more structured and effective approach to education and management for patients with T1D.

### Study Design

**Figure 1:**
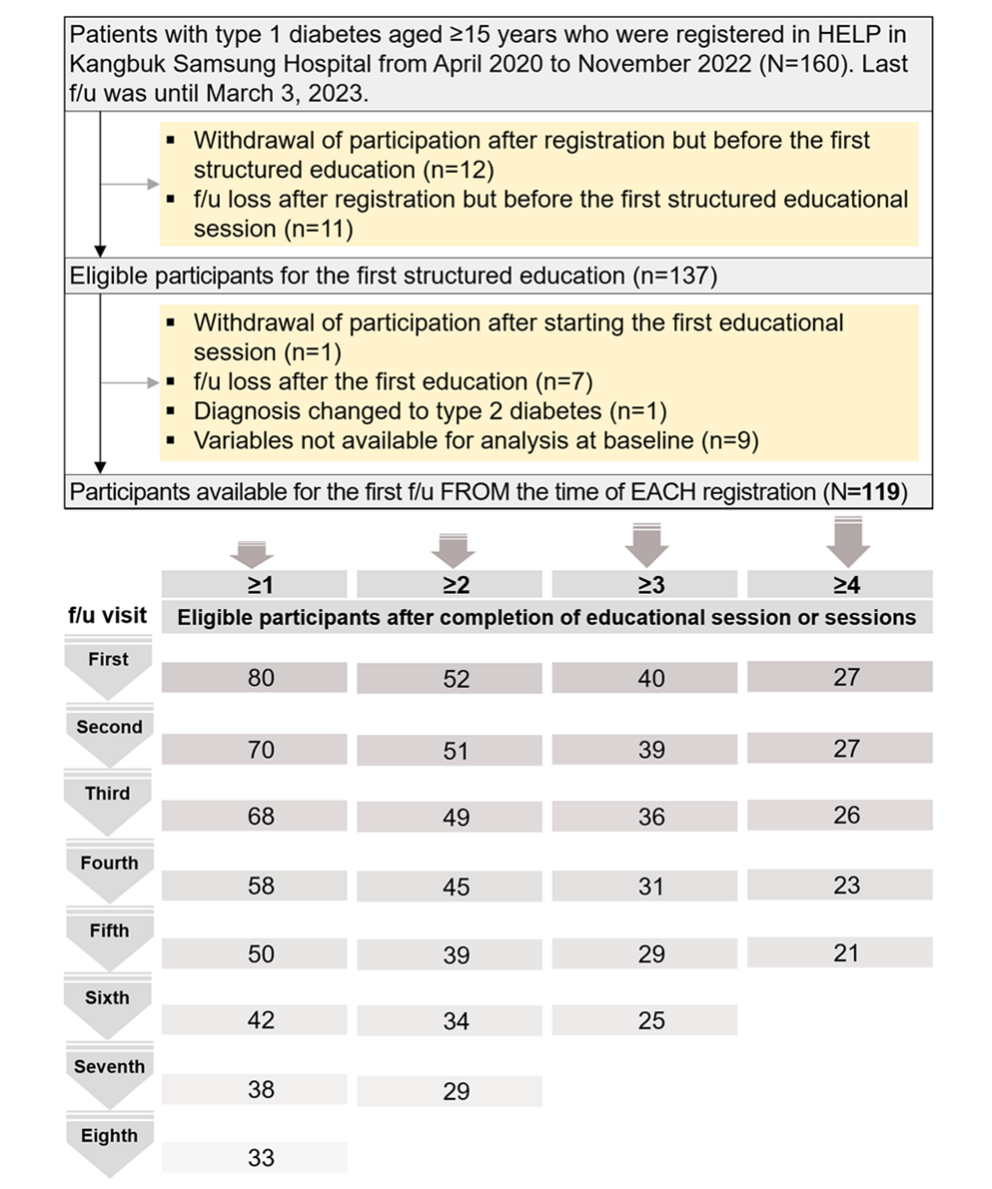
Diagram for study participants. The Home and Self-Care Program (HELP) means receiving at least 1 in-person structured education session (with remote digital support), which includes nursing and nutrition education as well as a physician consultation. f/u: follow-up.

This study is a real-world–based cohort study conducted from April 2020 to March 2023 in South Korea. The last data collection for this study occurred until March 3, 2023. The definition of T1D applied in this study was established according to the guidelines of the Health Insurance Review and Assessment Service under the Ministry of Health and Welfare of South Korea [11]. T1D was defined as 1 or more of the following: basal C-peptide of ≤0.6 ng/mL, stimulated C-peptide of ≤1.8 ng/mL, or 24-hour urine C-peptide of <3 µg per 24 hours; history of diabetic ketoacidosis at initial diagnosis; positive autoantibodies to pancreatic islets or insulin, such as the antiglutamic acid decarboxylase antibody; insulin administration required for proper glucose control; and disease code E10.x. During regular follow-up for each patient, the attending physician explained the purpose of HELP, and when the patient’s understanding and verbal consent were ascertained at the clinic, an experienced nurse or attending physician directly explained the consent form in additional detail. All participants were included if they met the age limit and were able to understand the program and receive educational sessions. Eligible participants were those who completed the required number of educational session or sessions, visited the outpatient clinic on the scheduled date, and provided complete data for statistical analysis (Figure 1).

### Structured Education

**Figure 2:**
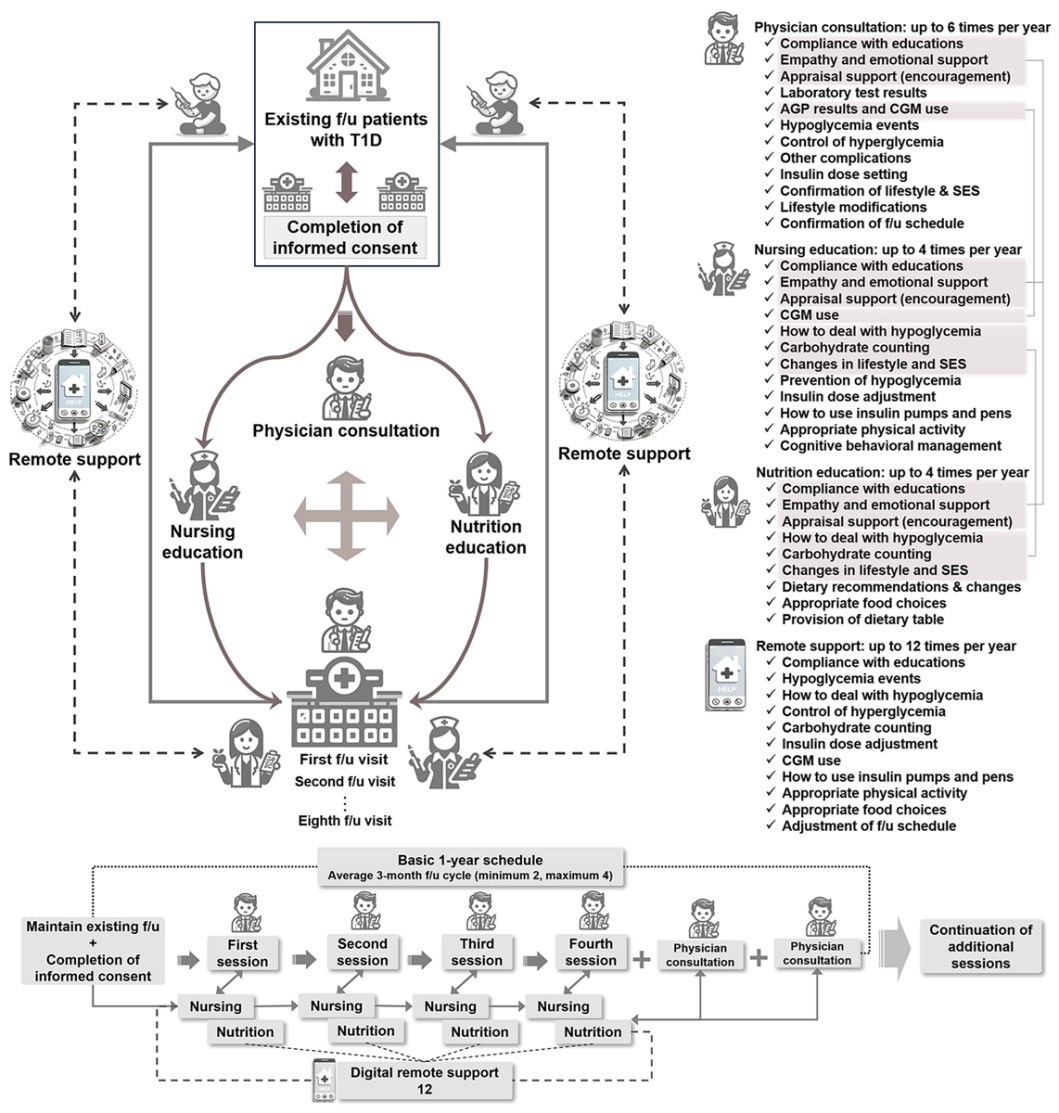
Schematic diagram for implementation of structured education sessions in the Home and Self-Care Program (HELP). HELP means receiving at least 1 structured education session (with remote digital support), which includes nursing and nutrition education as well as a physician consultation. AGP: ambulatory glucose profile; CGM: continuous glucose monitoring; f/u: follow-up; SES: socioeconomic status; T1D: type 1 diabetes.

The definition of receiving structured education (HELP) was that the participant received physician consultation, nursing education, and nutrition education at least once each. Participants consulted with a physician at every follow-up visit from the beginning up to 6 times per year. Nursing and nutrition education was provided separately up to 4 times per year at or between visits by experienced board-certified nurses and nutritionists. Each educational area included basic interventions that were applicable across all areas, along with specialized, individualized, and advanced intervention components specific to each area. Remote support encompassed all educational areas (Figure 2 and Multimedia Appendices 1 and 2).

The structured education described how to prevent and respond to hypoglycemia, control hyperglycemia, determine proper insulin dose control with carbohydrate counting, and optimally use and understand continuous glucose monitoring (CGM) for CGM users only. To induce overall lifestyle modification, dietary recommendations (including appropriate food choices and provision of a dietary table), exercise therapy, and cognitive behavioral management were also discussed. We also provided emotional support with empathy and appraisal support with encouragement. These educational contents were created based on the guidelines of the Korean Diabetes Association [12] and the Korean Society for the Study of Obesity [13] and were implemented in parallel with Kangbuk Samsung Hospital’s own nursing and food nutrition education programs already in progress. The individualized educational sessions were tailored to the characteristics and conditions of each patient.

In addition, this program is characterized by actively using remote support through smartphones to strengthen and sustain the educational effect after in-person education, which had not been attempted in previous structured education programs. All participants took part in this study using their own personal smartphones, and no separate equipment was provided. When the patients were outside the hospital, remote support with feedback on an existing general-purpose smartphone app (KakaoTalk Channel; Kakao Corp) was provided up to 12 times a year between education sessions. Through the channel established by Kangbuk Samsung Hospital, patients communicated based on SMS text messages, and when necessary, photos provided by patients were used as an element of communication. In most cases, patients initiated the request for help, and the medical staff responded with real-time or delayed answers depending on the situation. Medical staff may initiate communication first to confirm that the feedback has been correctly implemented by the patient. In addition, communication can also take place through video or phone calls. Remote support was not just for cases in which participants did not receive in-person education. This support was provided to ensure that participants were effectively applying what they had learned from the education, even if they had completed more than 1 session. In addition, in situations in which in-person education was delayed (eg, due to COVID-19 infection), remote support was provided to ensure continuity of education until the next education session. While in-person education is the basis of HELP, this constitutes additional support to enhance the effectiveness of the program.

### Primary Outcome

Participants were followed up on at average intervals of 3 months for up to 24 months. The timing of participation and the start of education varied for each patient, resulting in a different number of education sessions completed by participants during each follow-up period. We aimed to evaluate glycemic control status by follow-up visit according to the number of education sessions completed (Figure 1). We would like to emphasize that our study was based on a real-world setting. In general, most participants were observed based on the 3-month follow-up intervals, but some patients visited earlier than these intervals, whereas others visited at delayed intervals (Figure 2). Thus, we did not want to look at changes in glycated hemoglobin (HbA_1c_) levels over a specific period but rather wanted to check the degree of glycemic control (HbA_1c_) at the time of the follow-up visit after the education session or sessions. The key was to see whether glycemic control had improved at the next visit after receiving education session or sessions. The primary end point was the difference in mean HbA_1c_ values at each follow-up visit.

### Secondary Outcome

Before the start of the study, only a small number of participants (6/119, 5%) were using CGM consistently and properly, and we recommended CGM use after the start of the study, resulting in 83.2% (99/119) of the participants using CGM throughout the study period and being used for analysis. For study participants who were using CGM during the study period, we included CGM metrics as additional end points in our analysis using cloud-based ambulatory glucose profile (AGP), which was a time in the target range (TIR) of 70 to 180 mg/dL, time below the target range (TBR; <70 mg/dL and <54 mg/dL), and coefficient of variation (CV). It is emphasized that appropriate education in CGM use was added only for existing CGM users. CGM users were encouraged to actively use CGM (>70% of time CGM active) during the follow-up period rather than for a specific period. However, the percentage of time CGM is active varies significantly among CGM users. Most CGM users used the FreeStyle Libre 1 system, but some also used the Dexcom G5 or G6 and Guardian Connect systems.

### Data Collection

Information about each patient’s medical history and socioeconomic status (SES) was obtained from official medical charts and forms filled out by the patients. Blood pressure was measured at least twice using a standard sphygmomanometer in a sitting position with sufficient rest. Data were collected through a combination of medical chart reviews and patient-completed forms. Trained research assistants abstracted the data from the medical charts, ensuring consistency and accuracy in the process. Patients completed the forms independently, providing self-reported information on their health status and treatment experiences. The abstracted data were entered into a structured database designed for the study. To ensure data quality, we implemented several data checking and cleaning steps. This included verifying data entries against the original medical charts and patient forms, performing range and consistency checks, and resolving any discrepancies through a secondary review by another researcher. All biochemical tests were performed in laboratories using standardized equipment, and anthropometric measurements were taken by well-trained staff. Patients’ medical data were obtained at each visit.

CGM-related data were also obtained through cloud-based AGP at each follow-up visit after the first education session. As mentioned previously, HELP is defined as receiving in-person education, so patients who were supported only remotely due to delays in the visit cycle and unable to receive in-person education were excluded from the analysis of that follow-up cycle. CGM indexes (TIR, TBR, and CV) were confirmed from cloud-based AGP data. In some cases, AGP data could not be obtained intermittently during follow-up. In that case, they were excluded from the analysis related to that follow-up period. Blood glucose levels were assessed through the hexokinase method (Modular D2400; Hitachi). HbA_1c_ levels were measured using the immunoturbidimetric assay method (Cobra Integra 800 automatic analyzer; Roche Diagnostics). The urinary albumin-to-creatinine ratio was determined by measuring the spot urine albumin base in a radioimmunological competition assay (Immunotech) and the spot urine creatinine concentration using a modified Jaffe method.

### Statistical Analysis

The analysis was performed according to the number of structured educational sessions provided to participants. For continuous variables, appropriate representative values (mean or median) and spreads (SDs) are presented, and for categorical variables, frequencies and ratios are presented. A 2-tailed *t* test was conducted to compare continuous variables to assess differences in both directions, and the chi-square test was used to compare categorical variables. Paired *t* tests were used to compare HbA_1c_ levels and other values between baseline and each follow-up time point, with *P* values adjusted using Bonferroni correction for multiple testing. Regression analyses were performed to test the association between the number of education sessions and changes in HbA_1c_ levels and CGM metrics. The last observation carried forward imputation method was used for missing variables. Confounders were adjusted for, and the results are presented with 95% CIs for the primary end point—model 1 was adjusted for age, sex, baseline BMI, and baseline HbA_1c_, and model 2 was additionally adjusted for treatment type and diabetes mellitus duration. The supplemental analysis included variables such as educational level, social status, and employment status (Multimedia Appendix 3). CGM-related outcomes were adjusted for age, sex, BMI, HbA_1c_ level, treatment type, and duration of diabetes mellitus. All 2-tailed values with *P*<.05 were considered statistically significant. All statistical analyses were performed using R (version 4.3.2; R Foundation for Statistical Computing).

### Ethical Considerations

This study was reviewed and approved by the institutional review board of Kangbuk Samsung Hospital (KBSMC 202004028), Seoul, South Korea. Informed consent was obtained from all participants before their inclusion in the study for the primary data collection, including the provision that the collected data may be used for secondary analyses without obtaining additional consent from the participants. Although the HELP program was conducted prospectively, with all participants providing informed consent at the outset, research consent was not fully obtained from some participants. For these cases, we retrospectively analyzed the data with approval from the institutional review board. All procedures performed in this study were in accordance with the ethical standards of the institution. All study data were collected and stored in an anonymous format, ensuring that no personally identifiable information was included in the data set. No financial compensation was offered to participants. The study was conducted on a voluntary basis, and participants were informed of this before consenting to take part. Participants were informed of the significant impact that their participation would have on advancing our understanding of T1D.

## Results

### Study Participants

Participants in this study were selected from patients with T1D (aged ≥15 years). A total of 160 patients participated sequentially after completing the consent form. Patients who withdrew their participation (12/160, 7.5%) or were lost to follow-up (11/160, 6.9%) before the first structured education session were excluded. After the program started, a participant who withdrew their participation after the first physician consultation and before completing 1 education session (1/160, 0.6%), participants with complete loss to follow-up (7/160, 4.4%), a participant whose diagnosis changed to type 2 diabetes (1/160, 0.6%), and participants missing baseline variables (9/160, 5.6%) were further excluded from the analysis. Therefore, ultimately, 119 patients were available for study analysis at baseline (Figure 1).

### Characteristics of Participants

The median HbA_1c_ level of the study participants was 8.3% (IQR 7.3%-9.15%; mean 8.62%, SD 2.27%) at baseline, and the median duration of diabetes was 7.00 (IQR 2.00-13.00; mean 10.02, SD 16.10) years. Most of the study participants (110/119, 92.4%) controlled their glucose using multiple daily insulin injections (Table 1; Table S1 in Multimedia Appendix 3). After enrollment, most participants (99/119, 83.2%) checked their glucose using CGM. Although there was a slight difference, the BMI was statistically significantly lower in the group with relatively poor glycemic control (HbA_1c_≥8%). Among those who provided their SES information (63/119, 52.9%), participants with relatively better glycemic control (HbA_1c_<8%) had a relatively higher educational level (proportion of people with a bachelor’s degree or higher: 20/29, 69% with HbA_1c_<8% vs 10/34, 29% with HbA_1c_≥8%; *P*=.007; Table 1).

**Table 1:** Baseline characteristics of participants by glycated hemoglobin (HbA_c_) level (N=119)^a^.

Characteristic			Total participants	HbA_1c_ level			*P* value	
				<8% (n=50)	≥8% (n=69)			
Male participants, n (%)			61 (51.3)	20 (40)	41 (59.4)	.06		
Age (y), mean (SD)			45.84 (16.96)	43.98 (16.74)	47.19 (17.11)	.31		
**Age (y), n (%)**								.64
	<35		35 (29.4)	17 (34)	18 (26.1)			
	35-60		55 (46.2)	22 (44)	33 (47.8)			
	≥60		29 (24.4)	11 (22)	18 (26.1)			
**Duration of diabetes (y)**								
	Values, mean (SD)		10.02 (16.10)	12.08 (23.36)	8.52 (7.13)	.24		
	Values, median (IQR)		7.00 (2.00-13.00)	7.50 (2.00-13.00)	7.00 (2.00-12.00)	.94		
	**Subcategories, n (%)**							.80
		<3.0	33 (27.7)	14 (28)	19 (27.5)			
		3.0-10.0	37 (31.1)	17 (34)	20 (29)			
		≥10.0	49 (41.2)	19 (38)	30 (43.5)			
**HbA_1c_ level (%)**								
	Values, mean (SD)		8.62 (2.27)	6.95 (0.77)	9.84 (2.23)	<.001		
	Values, median (IQR)		8.30 (7.30-9.15)	7.20 (6.40-7.57)	9.00 (8.50-10.40)	<.001		
FBG^b^ (mg/dL), mean (SD)			146.97 (73.58)	132.02 (53.11)	158.11 (84.49)	.09		
PP2^c^ (mg/dL), mean (SD)			189.61 (90.86)	171.93 (93.77)	202.63 (88.91)	.35		
Body weight (kg), mean (SD)			61.86 (12.95)	64.27 (16.86)	60.15 (8.99)	.09		
BMI (kg/m^2^), mean (SD)			22.88 (3.60)	23.74 (4.78)	22.27 (2.31)	.03		
SBP^d^ (mm Hg), mean (SD)			119.98 (14.62)	122.27 (15.55)	118.39 (13.82)	.16		
DBP^e^ (mm Hg), mean (SD)			73.57 (10.67)	75.46 (9.77)	72.26 (11.14)	.11		
Creatinine (mg/dL), mean (SD)			0.78 (0.36)	0.77 (0.40)	0.79 (0.33)	.74		
eGFR^f^ (mL/min/1.73 m^2^), mean (SD)			99.69 (26.53)	98.13 (27.92)	100.81 (25.69)	.63		
uACR^g^ (mg/g), mean (SD)			80.70 (184.74)	84.44 (241.40)	78.25 (139.61)	.90		
**Type of insulin treatment, n (%)**								.88
	MDI^h^		110 (92.4)	46 (92)	64 (92.8)			
	Premixed insulin		3 (2.5)	1 (2)	2 (2.9)			
	Pump		6 (5)	3 (6)	3 (4.3)			
TDD^i^ of insulin (units), mean (SD)			45.90 (25.93)	46.80 (30.93)	45.25 (21.83)	.75		
**Socioeconomic status**								
	**Educational level, n (%)**							.007
		Primary school	3 (4.8)^j^	1 (3.4)^k^	2 (5.9)^l^			
		Middle or high school	30 (47.6)^j^	8 (27.6)^k^	22 (64.7)^l^			
		Bachelor’s degree or higher	30 (47.6)^j^	20 (69)^k^	10 (29.4)^l^			
	**Employment status, n (%)**							.10
		Active in work	28 (44.4)^j^	15 (51.7)^k^	13 (38.2)^l^			
		Retired or unemployed	9 (14.3)^j^	6 (20.7)^k^	3 (8.8)^l^			
		Other^m^	26 (41.3)^j^	8 (27.6)^k^	18 (52.9)^l^			
	**Social status, n (%)**							.58
		Married	27 (42.9)^j^	14 (48.3)^k^	13 (38.2)^l^			
		Other^n^	36 (57.1)^j^	15 (51.7)^k^	21 (61.8)^l^			
	**Caregiver, n (%)**							.47
		Self	18 (28.6)^j^	7 (24.1)^k^	11 (32.4)^l^			
		Domestic partner^o^	44 (69.8)^j^	22 (75.9)^k^	22 (64.7)^l^			
		Social support system	1 (1.6)^j^	0 (0)^k^	1 (2.9)^l^			
	**Income, n (%)**							.41
		Relatively low^p^	34 (54)^j^	15 (51.7)^k^	19 (55.9)^l^			
		Relatively high^q^	13 (20.6)^j^	8 (27.6)^k^	5 (14.7)^l^			
		No income or unknown	16 (25.4)^j^	6 (20.7)^k^	10 (29.4)^l^			

^a^Only 63 participants out of the total study population consented to provide their socioeconomic status information.

^b^FBG: fasting blood glucose.

^c^PP2: 2-hour postprandial glucose.

^d^SBP: systolic blood pressure.

^e^DBP: diastolic blood pressure.

^f^eGFR: estimated glomerular filtration rate.

^g^uACR: urine albumin-to-creatinine ratio.

^h^MDI: multiple daily injections.

^i^TDD: total daily dose.

^j^N=63.

^k^N=29.

^l^N=34.

^m^Housewives, students, and people with disabilities, among others.

^n^Unmarried, living alone, and widowed, among others.

^o^Spouse, parents, or descendants, among others.

^p^<5 million South Korean won (KRW) per month; assuming an average exchange rate of approximately 1200 KRW per US $ during the study period, the estimated amount is approximately US $4100 per month.

^q^≥5 million KRW per month.

### Primary Outcome

The HbA_1c_ reduction in participants who received structured educational session or sessions went from 1.63% (SD 2.03%; *P*<.001; after model 2 adjustment: 1.69%, 95% CI 1.24%-2.13%) at the first follow-up visit to 1.23% (SD 1.31%; *P*=.01; after model 2 adjustment: 1.28%, 95% CI 0.78%-1.79%) at the eighth follow-up visit after ≥1 session (Table 2) and from 1.74% (SD 2.45%; *P*=.01; after model 2 adjustment: 1.75%, 95% CI 0.72%-2.78%) at the first follow-up visit to 1.18% (SD 1.3%; *P*=.009; after model 2 adjustment: 1.17%, 95% CI 0.55%-1.85%) at the fourth follow-up visit after ≥4 sessions (Table S2 in Multimedia Appendix 3).

**Table 2:** Effects of the Home and Self-Care Program (HELP)^a^ on glycemic control in patients with type 1 diabetes (N=119).

Number of education sessions and follow-up visit^b^		Participants, n (%)	HbA_1c_^c^ level (%), mean (SD)		HbA_1c_ difference (%), mean (SD)	*P* value	Adjusted^d^ HbA_1c_ difference (%; 95% CI)
			At baseline	At follow-up visit			
≥**1**							
	First	80 (67.2)	8.54 (2.15)	7.33 (1.12)	1.63 (2.03)	<.001	1.69 (1.24-2.13)
	Second	70 (58.8)	8.53 (2.05)	7.37 (1.18)	1.67 (1.94)	.001	1.74 (1.27-2.20)
	Third	68 (57.1)	8.59 (2.10)	7.49 (1.38)	1.63 (2.01)	.002	1.69 (1.20-2.19)
	Fourth	58 (48.7)	8.58 (2.06)	7.41 (1.21)	1.65 (2.07)	.004	1.70 (1.13-2.27)
	Fifth	50 (42)	8.61 (2.11)	7.55 (1.09)	1.55 (1.98)	.02	1.66 (1.05-2.27)
	Sixth	42 (35.3)	8.70 (2.12)	7.65 (1.12)	1.51 (1.88)	.03	1.54 (0.92-2.17)
	Seventh	38 (31.9)	8.60 (1.99)	7.56 (1.17)	1.41 (1.66)	.02	1.44 (0.81-2.06)
	Eighth	33 (27.7)	8.48 (1.35)	7.54 (1.18)	1.23 (1.31)	.01	1.28 (0.78-1.79)

^a^HELP means receiving at least 1 structured education session (with remote digital support) comprising nursing and nutrition education and a physician consultation.

^b^Follow-up visit after the corresponding number of structured education session or sessions.

^c^HbA_1c_: glycated hemoglobin.

^d^Adjusted for age, sex, BMI, HbA_1c_ level, treatment type, and diabetes duration.

**Figure 3:**
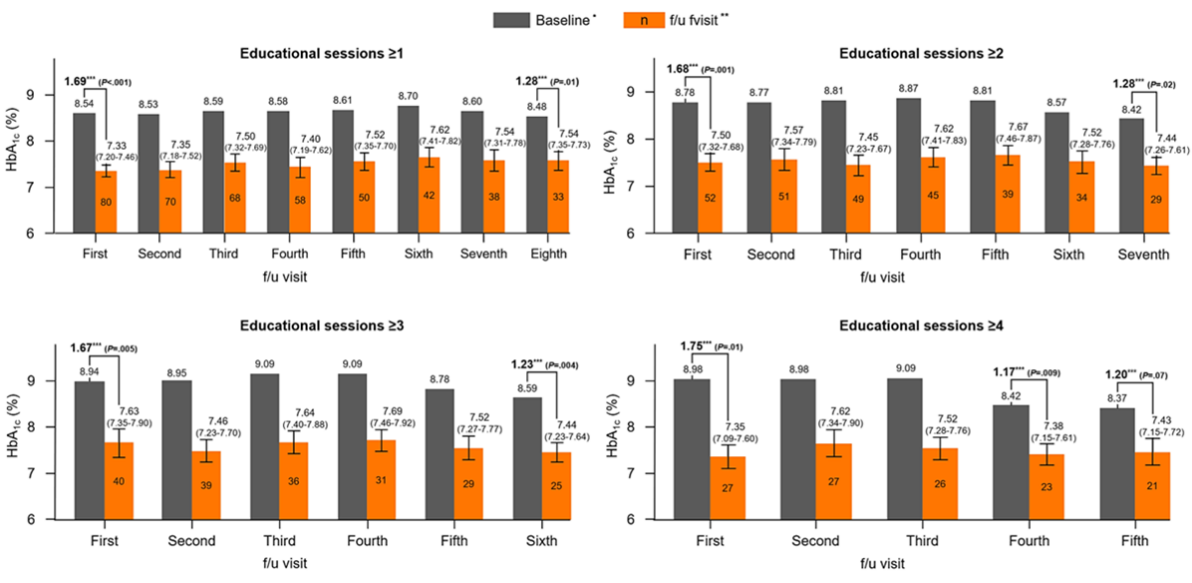
Improvement in glycated hemoglobin (HbA_1c_) levels after the Home and Self-Care Program (HELP) in patients with type 1 diabetes and its long-term maintenance. HELP means receiving at least one structured education session (with remote digital support), which includes nursing and nutrition education as well as a physician consultation. *Mean values at baseline. **Follow-up visit values adjusted for age, sex, BMI, HbA_1c_ level, treatment type, and diabetes duration (with 95% CIs). ***HbA_1c_ difference after adjustment. f/u: follow-up.

After full adjustment, the actual mean HbA_1c_ values remained between 7.33% (95% CI 7.2%-7.46%, at the first follow-up visit) and 7.62% (95% CI 7.41%-7.82%, at the sixth follow-up visit) after completing at least 1 session, and similarly remained between 7.35% (95% CI 7.09%-7.6%, at the first follow-up visit) and 7.62% (95% CI 7.34%-7.9%, at the second follow-up visit) after completing ≥4 sessions (Figure 3).

Even if no further education sessions were provided after the cessation of the program, the positive effect on glycemic control tended to be maintained for approximately 1 year, but the statistical significance was weak, and the number of participants in that analysis was small (Figure S1 and Table S3 in Multimedia Appendix 3).

In the analysis of participants who provided SES information, models adjusting for SES (ie, in the effort to exclude the effect of SES) showed a tendency for the degree of HbA_1c_ reduction to be more significant. This was particularly evident in patients who completed the educational sessions ≥2 times. However, when the number of sessions exceeded 4, the effect of SES decreased again (Table S4 in Multimedia Appendix 3). These effects were particularly pronounced in CGM users, especially in active users with a CGM activation time of 70% or more (Tables S5-S7 in Multimedia Appendix 3).

### Secondary Outcome

**Figure 4:**
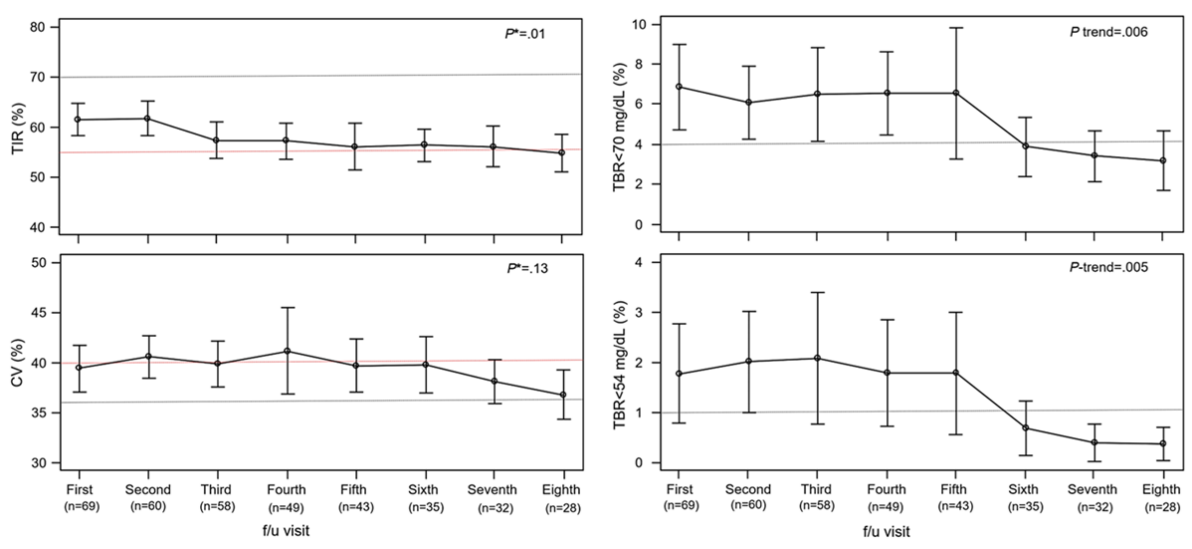
Long-term effects of the Home and Self-Care Program (HELP) on continuous glucose monitoring (CGM) metrics in patients with type 1 diabetes using CGM after at least one educational session (n=99 at baseline). All values at the follow-up visits were adjusted for age, sex, BMI, glycated hemoglobin level, treatment type, and diabetes duration and expressed with 95% CIs. HELP means receiving at least one structured education session (with remote digital support), which includes nursing and nutrition education as well as a physician consultation. *P value (in t test) for maintenance above the reference (time in the target range=55%) and below the reference (coefficient of variation [CV]=40%); TBR: time below the target range; TIR: time in the target range.

CGM metrics were officially recorded and analyzed after the first education session. The mean TIR values were maintained at >54.7% (corresponding to an HbA_1c_ level of approximately <7.6% [14])—from 61.77% (SD 16.79%; after adjustment: 61.59%, 95% CI 58.14%-65.03%) at the second follow-up visit to 55.85% (SD 20.61%; after adjustment: 54.7%, 95% CI 50.92%-58.48%) at the eighth follow-up visit after ≥1 session and from 59% (SD 13.72%; after adjustment: 59%, 95% CI 53.78%-64.22%) at the first follow-up visit to 55.86% (SD 14.91%; after adjustment: 55.92%, 95% CI 51.13%-60.72%) at the third follow-up visit after ≥4 sessions (Figure 4 and Table S8 and Figure S2 in Multimedia Appendix 3). The analysis of participants who provided SES information showed no significant differences according to SES (Table S9 in Multimedia Appendix 3).

The mean TBR gradually converged to within the recommended ranges (≤4% for TBR of <70 mg/dL and ≤1% for TBR of <54 mg/dL) [14] as follow-up progressed (Figure 4 and Table S8 and Figure S2 in Multimedia Appendix 3)—from 6.82% (SD 8.11%; after adjustment: 6.85%, 95% CI 4.72%-8.98%) at the first visit to 3.43% (SD 4.37%; after adjustment: 3.16%, 95% CI 1.68%-4.64%) at the eighth visit for a TBR of <70 mg/dL and from 1.76% (SD 3.48%; after adjustment: 1.77%, 95% CI 0.78%-2.77%) at the first visit to 0.44% (SD 0.89%; after adjustment: 0.36%, 95% CI 0.44%-0.89%) at the eighth visit after at least 1 session for a TBR of <54 mg/dL.

The mean CV value was maintained at <41.12% (41% for participants with SES information) after adjustment throughout the follow-up period (Figure 4 and Tables S8 and S9 and Figure S2 in Multimedia Appendix 3). These effects were also pronounced in active CGM users (>70% of “% time CGM active”; Table S10 in Multimedia Appendix 3).

## Discussion

### Principal Findings

The aim of this study was to develop a structured education program for patients with T1D that was feasible in the real world and imposed no additional cost burden on patients. We tried to demonstrate that, by individualizing and expanding existing in-person education and support programs, their effects can be significantly amplified and prolonged with simple additional remote support. This study provides evidence that HELP is clinically effective in improving glycemic control in patients with T1D (see Multimedia Appendix 4 for a graphical summary). Mean HbA_1c_ values decreased by 1.63% (after adjustment: 1.69%) at the first follow-up visit after completing only 1 education session. This effect was higher among CGM users (1.69% [after adjustment: 1.78%] for all CGM users and 2.02% [after adjustment: 2.05%] for active CGM users with >70% of “% time CGM active”). As the follow-up period progressed, the degree of reduction tended to gradually decline, but an overall decrease in HbA_1c_ levels of at least 1.2% was maintained for the entire follow-up period (approximately 24 months). In addition, although statistical significance was weak, the reduction in mean HbA_1c_ level tended to be sustained for approximately 12 months even with only simple follow-up after discontinuing the program. These results imply that, even after the educational program ends, patients keep using the knowledge and skills provided to them.

It is interesting to note that the reduction in HbA_1c_ levels did not continue to increase significantly when the number of structured educational sessions increased. When confounding factors such as SES, including educational background, were adjusted for and the effect of the structured education itself was clarified, the degree of HbA_1c_ level reduction increased slightly as the number of educational sessions increased. However, this pattern disappeared in patients who received >4 educational sessions. These findings require some careful consideration. Those who received fewer educational sessions had lower mean HbA_1c_ levels at baseline, suggesting that their glycemic control potential and educational compliance were likely to be relatively good. Therefore, we presume that even after only 1 educational session, the glucose-lowering effect can be maintained with simple follow-up without the need for additional sessions. In contrast, those who received more educational sessions had higher mean baseline HbA_1c_ levels, suggesting that they were likely having difficulty controlling their glucose. Those patients might need more educational sessions and longer follow-up periods to achieve effects similar to those in the former group.

Another important clinical outcome in this study was that hypoglycemia was not exacerbated despite the overall glycemic reduction; instead, the hypoglycemia indexes also improved gradually. The assessment of hypoglycemia events was based on TBR data from the AGPs due to the scarcity and irregularity of self-monitoring of blood glucose results. In a TBR of both <70 mg/dL and <54 mg/dL, the pattern of converging within the recommended level (4% and 1%, respectively) [14] was clear as the education sessions and follow-up visits progressed. It is interesting that both indexes fell below the recommended levels at similar times, likely due to the gradual appearance of preventive effects against hypoglycemia after education. CV (recommended level: <36% [14]) is an indicator of glycemic variability and presents many complexities because it is influenced not only by TIR and TBR but also by the time above the range [14]. In this study, mean CV values remained below the maximum of 42.1% (41.1% after adjustment) throughout the follow-up period and did not change significantly. However, unlike TIR, CV appeared to correlate somewhat with the improvement patterns in TBR as the number of education sessions and follow-up visits increased. In this study, we suspect that the gradually and significantly improved TBR had a greater impact on the change in CV values than TIR (which remained relatively stable despite a slight gradual decrease).

### Comparison With Prior Work

The key point we wish to emphasize in our study is that, by adapting and integrating existing in-person educational support programs into a digital communication app, it is possible to achieve long-term glycemic control outcomes in patients with T1D that have not been realized in previous studies. This approach is not only immediately applicable in clinical settings but also highly effective, using widely available smartphone communication apps with minimal cost and resources. This approach could serve as a robust model for digital remote support programs for patients with diabetes that are being promoted by the Korean government.

We aimed to develop comprehensive content and protocols that would maximize the benefits reported in previous studies. The unique advantage of our study compared with previous studies on similar educational programs is that it not only consisted of 1-time in-person education but was also supplemented with remote support by using an existing general-purpose smartphone app to strengthen and sustain the educational effect. It is also true that efforts were made to personalize the usability of the app. Because the ability to understand education and use smartphone apps varies depending on conditions such as age, gender, occupation, and academic ability, our medical staff adjusted the level of education according to the patient’s level of understanding and used the method preferred by the patient (text, photo, video, or call).

Our research stands out for its practical integration of structured education and remote support using a common smartphone communication app, which is both cost-effective and scalable. Studies such as the “bant” app trial [15] show promise but are often limited by user engagement challenges, whereas app cloud systems [16] demonstrate modest gains in self-management behaviors without substantial HbA_1c_ level reductions. Unlike other studies [15-17] that may show limited long-term engagement or modest clinical outcomes, our study demonstrated sustained glycemic control improvements over time. This long-term effect and the seamless integration into real-world clinical settings make our approach highly applicable and potentially more impactful in broader health care systems, especially as an economical prototype for government-supported telemedicine programs.

It has already been established by many previous studies that promoting the actual or potential self-management capabilities of patients with diabetes is essential for improving short- and long-term prognosis regardless of the type of education used [18]. The implementation of a structured education program and follow-up for 6 months to 1 year simultaneously improved mean HbA_1c_ values and QOL without worsening hypoglycemia [19,20]. The effects of structured education for intensive insulin treatment in reducing mean HbA_1c_ values and improving hypoglycemia or QOL are already well known [21-23]. Moreover, nutritional education, including carbohydrate counting, has been shown to be beneficial in improving HbA_1c_ levels and increasing the consumption of recommended foods, although no robust evidence indicates that it improves glycemic control in patients with T1D yet [24-26]. Although the content of the education sessions offered and the characteristics of the study participants differ, those results are consistent with our research results.

Many previous studies have investigated the benefits of CGM and found that it provides significant improvement in the hypoglycemic index (TBR) and mean glucose values (eg, HbA_1c_ or TIR) of patients with T1D only when appropriately structured education is provided [27-29]. Most of our participants in this study were using CGM (99/119, 83.2%), and those CGM users showed better results than other patients, especially active CGM users. It is likely that the effects of structured education on them influenced the overall positive outcomes of this study. Because the TIR value could not be obtained from baseline, we attempted to confirm the patterns of change in TIR according to the number of education sessions beginning at the first follow-up visit. The recommended TIR value for patients with T1D using CGM is >70% [14]. Although it varies by study, HbA_1c_ values of 7.4% (95% CI 6.1%-8.8%) and 7.9% (95% CI 6.6%-9.2%) generally correspond to a TIR of 60% and 50%, respectively [14]. The participants in this study who completed at least 1 educational session maintained a mean TIR of ≥55% (HbA_1c_ of approximately ≤7.6%) during the entire follow-up period. It is noteworthy that these outcomes are slightly better than the results of previous studies that compared glycemic control between patients using CGM and capillary blood measurement [30,31]. We confirmed that appropriate consistency was maintained between the mean HbA_1c_ values measured in this study and the mean TIR values in the AGP data of the study participants. The pattern of change in TIR values was similar to that of HbA_1c_ values, being relatively constant throughout the follow-up period without significant changes.

One more interesting finding in this study was the association between educational level and glycemic control and CGM use. Of the total study participants, 52.9% (63/119) provided their educational background. Among them, the group with relatively good glycemic control (HbA_1c_<8%) at baseline had a higher proportion of individuals with a bachelor's degree or higher compared to the other group (n=30: 20/29, 69% vs 10/34, 29%; *P*=.007; Table 1). It has been suggested that the level of education may be related to complication awareness and treatment compliance in patients with type 2 diabetes [32] and that overall SES is correlated with glycemic control and complications in patients with T1D using flash glucose monitoring [33]. Although that was not the main concern of our study, it might have had some influence on the results. Because schooling information was available for only approximately half of our participants, caution is needed in interpretation. The data in Multimedia Appendix 3 adjust the results for SES-related information.

### Limitations

This study has some limitations. Even though it is prospective, cohort studies have inherent limitations. Because it was not a randomized controlled trial, patients with high compliance might have been selectively recruited, and many confounding factors might have influenced the results despite our adjustment efforts. A high level of attrition (loss to follow-up) could lead to biased results. We have no objective verification of patient-provided information. Not all participants were able to properly provide their SES, including their educational level and economic status. Hypoglycemic events could not be confirmed through serum or capillary blood test, so cloud-based AGP data had to be used. CGM activity varied widely among patients using CGM, so the consistency of the AGP data could be limited. Different manufacturers and brands of CGM used by patients may also have some limitations. HbA_1c_ is an indicator of a 2- to 3-month average of blood glucose levels. Most participants were observed based on the 3-month follow-up intervals, but some patients visited earlier than these intervals, whereas others visited at delayed intervals. We tried to reconfirm the consistency of the presented HbA_1c_ values by observing the TIR values (reflecting the average HbA_1c_ in CGM users) and continuously tracking the patients’ sequential HbA_1c_ values over a long period. Finally, despite the unfavorable conditions for glycemic control posed by the COVID-19 pandemic, paradoxically, presenting good results in this study can be considered a strength. However, the pandemic may have influenced some of the intervention processes applied in this study, which could be a limitation in interpreting the results.

### Conclusions

Despite some limitations, this study has clinical and policy significance because it is the first study to implement and clinically confirm the positive results of a home health care project for patients with T1D supported by the Korean government. The periodic implementation of HELP has been demonstrated to be very effective in improving both the short- and long-term glycemic control of patients with T1D. Our task now is to further simplify the program to enable 1-stop educational support and restructure the content so that even patients with lower educational levels can easily adhere to it. To increase the use of remote support, it will be necessary to secure dedicated medical staff and use simulations at the beginning of the education sessions in a hospital. By strengthening the reliability of these results through more objective studies, such as randomized controlled trials, we expect to facilitate the general application of HELP. In addition, future research could explore the interesting comparison between cases in which only in-person education is provided and cases in which remote support is provided alongside in-person education.

## Appendix

Multimedia Appendix 1 Home patient review checklist.

Multimedia Appendix 2 Basic nutritional education course.

Multimedia Appendix 3 Supplementary tables and figures.

Multimedia Appendix 4 Graphical abstract.

## Abbreviations

AGP: ambulatory glucose profile
CGM: continuous glucose monitoring
CV: coefficient of variation
HbA_1c_: glycated hemoglobin
HELP: Home and Self-Care Program
QOL: quality of life
SES: socioeconomic status
T1D: type 1 diabetes
TBR: time below the target range
TIR: time in the target range

## Data Availability

The data sets generated and analyzed during this study are available from the corresponding author on reasonable request.
