# Supplementary material for: Long-Term Glycemic Control Improvement After the Home and Self-Care Program for Patients With Type 1 Diabetes: Real-World–Based Cohort Study
Source: J Med Internet Res. 2024 Sep 11;26:e60023. doi: 10.2196/60023 (PMC11425018; doi:10.2196/60023)
Supplement: Multimedia Appendix 1 [file jmir_v26i1e60023_app1.pdf]

Checklist for Patient Review at Home

Current HbA1c: ____%/ Last HbA1c: _____%/ TDD: _______u/ ISF: _______/ ICR: _______.
CGM Brand: _______________/ TIR: _____%/ TBR: _____%(70), _____%(54)/ CV: _____%.

Basic Information:

- Insulin Type and Administration Method: Ensure appropriateness.
- CGM (Continuous Glucose Monitor) Usage: Check insulin administration records and input.
- CGM Device Suitability: Ensure appropriate device and application site.
- Hypoglycemia Management: Verify appropriateness:
  - Check SMBG (Self-Monitoring of Blood Glucose) value for hypoglycemia symptoms.
  - After intervention, check SMBG value, not CGM.
  - Ensure no repeated hypoglycemia or hyperglycemia due to improper management.
  - Educate about the principle of time lag.

Blood Glucose Pattern Check:

- Pre-meal Hypoglycemia: Check:
  - Diet: Protein deficiency, lower carbohydrate intake.
  - Increased activity post previous meal.
- Dawn/Pre-sleep Hypoglycemia: Verify:
  - Provide pre-sleep snack advice.
- Post-meal Hyperglycemia: Check:
  - Meal timing, order of intake, high GI (Glycemic Index) diet, post-meal activity.
- Post-meal Blood Glucose Fluctuations: Check:
  - Diet: Only protein with insulin, high-protein/high-fat diet without carbohydrates.
  - Indigestion and prolonged meal time.
- Late Post-meal Hyperglycemia: Verify:
  - High-fat/high-protein diet, overeating.

Insulin Dosage Adjustment & Setting Verification:

- Post-meal Hyperglycemia: Check additional dosage:
  - Timing (2-4 hours), trend arrows, IOB (Insulin on Board) consideration.
- Insulin Administration Timing:
  - Interval with meal: Immediate hyperglycemia if too early, immediate hypoglycemia if too late.
- High-fat/High-protein Diet Additional Dosing Education:
  - Use IFR (Insulin-to-Fat Ratio) with NPH insulin timing (refer to chart).
  - Hold additional correction within 2-4 hours after meal.
- Carbohydrate Counting Appropriateness:
- ICR (Insulin-to-Carbohydrate Ratio) Appropriateness:
  - Ensure first meal accuracy, compare pre- and post-meal blood glucose within ±30.
- ISF (Insulin Sensitivity Factor) Appropriateness:
- Titration Method Check:
  - Basal insulin adjustment: Reduce if hypoglycemia occurs more than once overnight, increase if hyperglycemia exceeds 25%.
  - Meal insulin adjustment: Apply ISF with a very sensitive scale (pre-meal target 80-140).
  - Verify basic dose setting with ICR and additional dose with ISF.

Additional Considerations:

- Trend Arrow Understanding:

- Exercise Timing and Type/Intensity:
  - Initial advice: 30 minutes to 1 hour post-meal.
  - Hypoglycemia during exercise: Reduce rapid-acting insulin by 20-30% pre-exercise and monitor.
  - Excessive exercise: Test and record adjustments.
- Stable Calibration Timing:
- Data (AGP - Ambulatory Glucose Profile) Review Education:
- Meal App Usage Confirmation:

Pump Patient Additional Education:
- Infusion Set/Reservoir Replacement Suitability: Verify replacement frequency and cannula/tube filling volume.
- Insulin Type and AIT (Active Insulin Time) Suitability:
- Target Blood Glucose Setting Appropriateness:
- Basal Insulin Timing Dose Appropriateness:
- Bolus Calculator Usage Appropriateness:
- Post-meal Automatic Correction Bolus Pattern Check:
- Temporary Basal or Target Value Usage and Education:
- Daily Total, Basal/Bolus Ratio, Meal/Auto-correction Ratio:
- Data (AGP) Review Education:
- Disconnect Timing and Sensor Replacement Blood Glucose Pattern Check:
- Shower Disconnect Hyperglycemia Pattern and Additional Dosage:

Self-Problem List:

Mentioned During Last Education:

Other Special Notes:
