# Supplementary figures and images for: Long-Term Glycemic Control Improvement After the Home and Self-Care Program for Patients With Type 1 Diabetes: Real-World–Based Cohort Study
Source: J Med Internet Res. 2024 Sep 11;26:e60023. doi: 10.2196/60023 (PMC11425018; doi:10.2196/60023)

# Nutritional Education for Home and Self-care Program for Type 1 Diabetic Patients

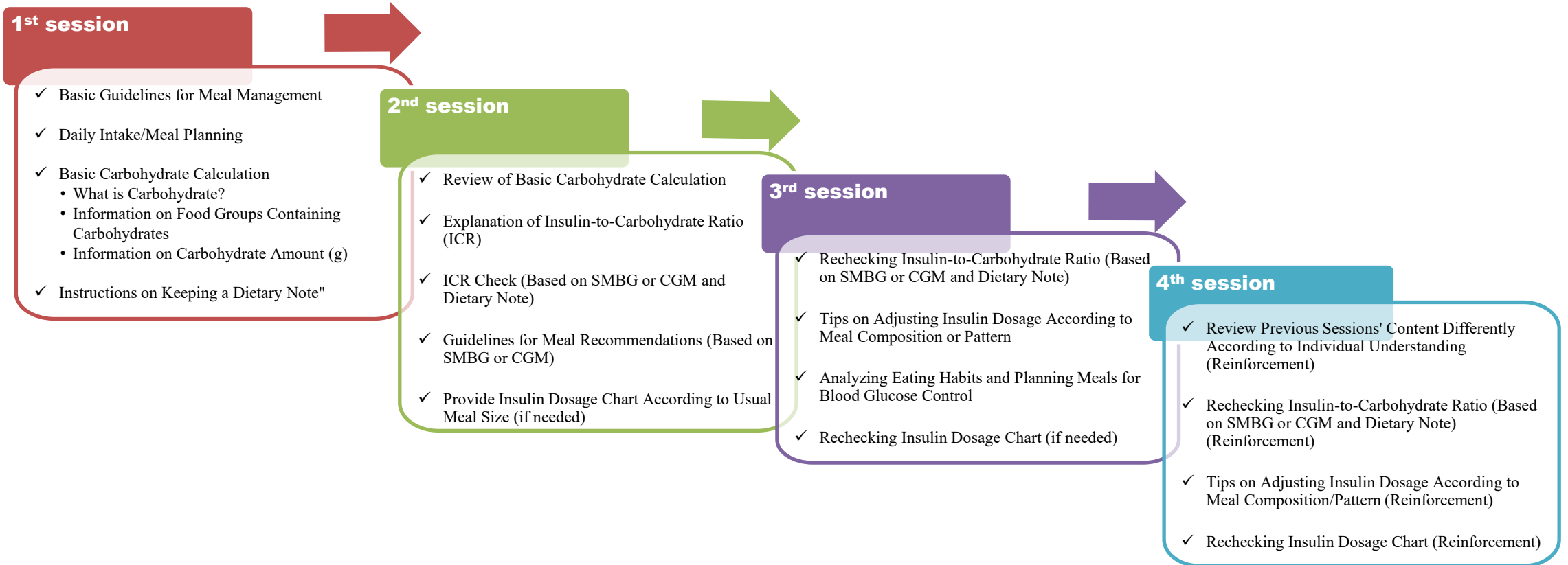

Supplement: Multimedia Appendix 2 [file jmir_v26i1e60023_app2.pdf]
