# Supplementary material for: Long-Term Glycemic Control Improvement After the Home and Self-Care Program for Patients With Type 1 Diabetes: Real-World–Based Cohort Study
Source: J Med Internet Res. 2024 Sep 11;26:e60023. doi: 10.2196/60023 (PMC11425018; doi:10.2196/60023)
Supplement: Multimedia Appendix 3 [file jmir_v26i1e60023_app3.pdf]

This is a Multimedia Appendix 3 to a full manuscript published in the J Med Internet Res. For full copyright and citation information see 'Long-Term Glycemic Control Improvement After the Home and Self-Care Program for Patients With Type 1 Diabetes: Real World–Based Cohort Study'.

Supplementary Tables

Table S1. Baseline Characteristics of participants according to CGMa use after enrollment.

Table S2. Effects of HELP[a] on glycemic control in patients with T1D[b].

Table S3. Residual effects of HELP[a] on glycemic control after cessation of educations in patients with T1D[b] (n = 34).

Table S4. Effects of HELP[a] on glycemic control in T1D[b] patients with SES[c] information (n = 63).

Table S5. Effects of HELP[a] on glycemic control in CGM[b] users (n = 99).

Table S6. Effects of HELP[a] on glycemic control in CGM[b] users with SES[c] information (n = 53).

Table S7. Effects of HELP[a] on glycemic control in CGM[b] users with '% time CGM active' of ≥70%.

Table S8. Effects of HELP[a] on CGM[b] metrics in patients with T1D[c] (n = 99).

Table S9. Effects of HELP[a] on CGM[b] metrics in T1D[c] patients with SES[d] information (n = 53).

Table S10. Effects of HELP[a] on CGM[b] metrics in T1D[c] patients with '% time CGM active' of ≥70%.

Supplementary Figures

Figure S1. Residual effects of HELP[a] on glycemic control in patients with T1D[b] after cessation of education.

Figure S2. Long term effects of HELP[a] on CGM[b] metrics in patients with T1D[c]. TIR[d] (A), TBR[e] <70 mg/dL (B), TBR <54 mg/dL (C), and CV[f] (D).

Table S1. Baseline Characteristics of participants according to CGM[a] use after enrollment.

Characteristics	Total	CGM		*P*
		Non-user	User	
No. of subjects, n (%)	119	20 (16.8%)	99 (83.2%)	
Male, n (%)	61 (51.3)	12 (60.0)	49 (49.5)	.54
Age, year	45.84 (±16.96)	49.05 (±16.06)	45.19 (±17.14)	.36
Subcategories, n (%)				.57
< 35.0	35 (29.4)	4 (20.0)	31 (31.3)	
35.0 - 60.0	55 (46.2)	10 (50.0)	45 (45.5)	
≥ 60.0	29 (24.4)	6 (30.0)	23 (23.2)	
Diabetes duration, mean, year	10.02 (±16.10)	10.00 (±9.49)	10.02 (±17.16)	.99
Diabetes duration, median, year	7.00 [2.00, 13.00]	7.00 [2.00, 15.25]	7.00 [2.00, 13.00]	.63
Subcategories, n (%)				.97
< 3.0	33 (27.7)	6 (30.0)	27 (27.3)	
3.0 - 10.0	37 (31.1)	6 (30.0)	31 (31.3)	
≥ 10.0	49 (41.2)	8 (40.0)	41 (41.4)	
HbA1c[b], mean, %	8.62 (±2.27)	8.79 (±2.56)	8.59 (±2.22)	.72
HbA1c, median, %	8.30 [7.30, 9.15]	8.00 [7.62, 8.62]	8.30 [7.25, 9.20]	.95
FBG[c], mg/dl	146.97 (±73.58)	129.07 (±65.20)	150.28 (±74.92)	.31
PP2[d], mg/dl	189.61 (±90.86)	160.00 (±98.64)	199.08 (±88.23)	.30
Body weight, kg	61.86 (±12.95)	61.19 (±14.55)	61.99 (±12.68)	.80
BMI[e], kg/m^2	22.88 (±3.60)	22.33 (±4.01)	22.99 (±3.53)	.45
SBP[f], mmHg	119.98 (±14.62)	126.90 (±20.07)	118.56 (±12.90)	.02
DBP[g], mmHg	73.57 (±10.67)	79.10 (±12.67)	72.43 (±9.91)	.01
Creatinine, mg/dL	0.78 (±0.36)	0.69 (±0.18)	0.80 (±0.38)	.28
eGFR[h], mL/min/1.73m^2	99.69 (±26.53)	95.07 (±34.06)	100.72 (±24.70)	.43
uACR[i], mg/g	80.70 (±184.74)	129.94 (±173.48)	69.18 (±187.17)	.33
Type of insulin treatment, n (%)				.40
MDI[j]	110 (92.4)	19 (95.0)	91 (91.9)	
Premixed insulin	3 (2.5)	1 (5.0)	2 (2.0)	
Pump	6 (5.0)	0 (0.0)	6 (6.1)	
TDD[k] of insulin, unit	45.90 (±25.93)	50.75 (±28.61)	44.92 (±25.40)	.36
Schooling, n (%)				.40
Primary school	3 (4.8)	1 (10.0)	2 (3.8)	
Middle or high school	30 (47.6)	6 (60.0)	24 (45.3)	
Bachelor's degree or higher	30 (47.6)	3 (30.0)	27 (50.9)	
Employment status, n (%)				.27
Active in work	28 (44.4)	3 (30.0)	25 (47.2)	
Retired or unemployed	9 (14.3)	3 (30.0)	6 (11.3)	
Other 1[l]	26 (41.3)	4 (40.0)	22 (41.5)	
Social status, n (%)				.99
Married and with spouse	27 (42.9)	4 (40.0)	23 (43.4)	
Other 2[m]	36 (57.1)	6 (60.0)	30 (56.6)	
Caregiver, n (%)				.91
Self	18 (28.6)	3 (30.0)	15 (28.3)	
Domestic partners[n]	44 (69.8)	7 (70.0)	37 (69.8)	
Social support system	1 (1.6)	0 (0.0)	1 (1.9)	
Income, n (%)				.43

Relatively low[o]	34 (54.0)	6 (60.0)	28 (52.8)
Relatively high[p]	13 (20.6)	3 (30.0)	10 (18.9)
No income or unknown	16 (25.4)	1 (10.0)	15 (28.3)

Values are expressed as n (%), mean (± standard deviation), median [interquartile range], or percentage.

Only 63 participants out of the total study population consented to provide their socioeconomic status information.

[a] CGM: continuous glucose monitoring.

[b] HbA1c: hemoglobin A1c.

[c] FBG: fasting blood glucose

[d] PP2: postprandial 2 hours glucose

[e] BMI: body mass index.

[f] SBP: systolic blood pressure

[g] DBP: diastolic blood pressure

[h] eGFR: estimated glomerular filtration rate

[I] uACR: urine albumin-creatinine ratio

[j] MDI: multiple daily injections

[k] TDD: total daily dose

[l] other 1: housewives, students, disabled people, etc.

[m] other 2: unmarried, living alone, widowed, etc.

[n] domestic partners: spouse, parents, descendants, etc.

[o] $< 5 \times 10^6$ South Korean Won (KRW)/month
: assuming an average exchange rate of approximately 1,200 KRW per US dollar (USD) during the study period, the estimated amount is roughly 4,100 USD per month."

[p] $\geq 5 \times 10^6$ KRW/month

Table S2. Effects of HELP[a] on glycemic control in patients with T1D[b].

No. of education session	f/u visit[c]	Subjects	HbA1c[d] (%)		HbA1c difference, %	*P*	HbA1c difference, 95% CI	
			at baseline	at f/u visit			Model 1[e]	Model 2[f]
≥ 1	1st	80	8.54 ± 2.15	7.33 ± 1.12	1.63 ± 2.03	< .001	1.67 (1.22 - 2.13)	1.69 (1.24 - 2.13)
	2nd	70	8.53 ± 2.05	7.37 ± 1.18	1.67 ± 1.94	.001	1.72 (1.25 - 2.19)	1.74 (1.27 - 2.20)
	3rd	68	8.59 ± 2.10	7.49 ± 1.38	1.63 ± 2.01	.002	1.67 (1.17 - 2.17)	1.69 (1.20 - 2.19)
	4th	58	8.58 ± 2.06	7.41 ± 1.21	1.65 ± 2.07	.004	1.67 (1.09 - 2.25)	1.70 (1.13 - 2.27)
	5th	50	8.61 ± 2.11	7.55 ± 1.09	1.55 ± 1.98	.02	1.62 (1.01 - 2.24)	1.66 (1.05 - 2.27)
	6th	42	8.70 ± 2.12	7.65 ± 1.12	1.51 ± 1.88	.03	1.52 (0.90 - 2.14)	1.54 (0.92 - 2.17)
	7th	38	8.60 ± 1.99	7.56 ± 1.17	1.41 ± 1.66	.02	1.42 (0.81 - 2.03)	1.44 (0.81 - 2.06)
	8th	33	8.48 ± 1.35	7.54 ± 1.18	1.23 ± 1.31	.01	1.28 (0.78 - 1.77)	1.28 (0.78 - 1.79)
≥ 2	1st	52	8.78 ± 2.00	7.52 ± 1.24	1.62 ± 1.97	.001	1.64 (1.06 - 2.22)	1.68 (1.10 - 2.26)
	2nd	51	8.77 ± 2.02	7.55 ± 1.24	1.63 ± 1.96	.002	1.63 (1.03 - 2.23)	1.68 (1.09 - 2.27)
	3rd	49	8.81 ± 2.05	7.45 ± 1.03	1.57 ± 2.00	< .001	1.58 (0.96 - 2.19)	1.64 (1.03 - 2.24)
	4th	45	8.87 ± 2.08	7.66 ± 1.08	1.61 ± 2.07	.008	1.66 (0.98 - 2.34)	1.72 (1.04 - 2.40)
	5th	39	8.81 ± 2.14	7.69 ± 1.13	1.52 ± 1.95	.02	1.54 (0.87 - 2.20)	1.57 (0.90 - 2.24)
	6th	34	8.57 ± 1.99	7.49 ± 1.21	1.51 ± 1.70	.03	1.49 (0.85 - 2.14)	1.51 (0.85 - 2.17)
	7th	29	8.42 ± 1.29	7.45 ± 1.14	1.25 ± 1.37	.02	1.27 (0.72 - 1.82)	1.28 (0.71 - 1.84)
≥ 3	1st	40	8.94 ± 2.13	7.62 ± 1.25	1.62 ± 2.12	.005	1.60 (0.88 - 2.32)	1.67 (0.95 - 2.39)
	2nd	39	8.95 ± 2.16	7.48 ± 0.96	1.60 ± 2.13	.001	1.62 (0.88 - 2.35)	1.69 (0.96 - 2.41)
	3rd	36	9.09 ± 2.19	7.67 ± 1.04	1.69 ± 2.27	.008	1.72 (0.89 - 2.55)	1.80 (0.96 - 2.63)
	4th	31	9.09 ± 2.26	7.71 ± 1.01	1.58 ± 2.15	.01	1.58 (0.80 - 2.36)	1.61 (0.83 - 2.39)
	5th	29	8.78 ± 2.08	7.52 ± 1.11	1.54 ± 1.78	.01	1.53 (0.80 - 2.25)	1.54 (0.79 - 2.29)
	6th	25	8.59 ± 1.29	7.43 ± 1.05	1.25 ± 1.41	.004	1.24 (0.64 - 1.83)	1.23 (0.62 - 1.85)
≥ 4	1st	27	8.98 ± 2.28	7.35 ± 0.91	1.74 ± 2.45	.01	1.74 (0.71 - 2.77)	1.75 (0.72 - 2.78)
	2nd	27	8.98 ± 2.28	7.63 ± 1.10	1.62 ± 2.27	.04	1.62 (0.70 - 2.55)	1.63 (0.67 - 2.59)
	3rd	26	9.00 ± 2.31	7.51 ± 1.01	1.59 ± 2.24	.02	1.61 (0.67 - 2.55)	1.59 (0.62 - 2.57)
	4th	23	8.42 ± 1.08	7.37 ± 1.01	1.18 ± 1.30	.009	1.18 (0.61 - 1.74)	1.17 (0.58 - 1.77)
	5th	21	8.37 ± 1.12	7.43 ± 0.98	1.21 ± 1.41	.07	1.20 (0.60 - 1.81)	1.20 (0.55 - 1.85)

Values are expressed as n (%), mean ± standard deviation, percentage, or median (interquartile range).

[a] Home and Self-care Program (HELP) means receiving at least one structured education session (with remote digital support) comprising nursing and nutrition education and a physician consultation.

[b] T1D: type 1 diabetes.

[c] Follow-up visit after the corresponding number of structured educations session(s).

[d] HbA1c: hemoglobin A1c

[e] Model 1: adjusted for age, sex, body mass index, and HbA1c.

[f] Model 2: Model 1 + additional adjustment for treatment type and diabetes duration.

Table S3. Residual effects of HELP[a] on glycemic control after cessation of educations in patients with T1D[b] (n = 34).

f/u visit[c]	Subjects	No. of education			HbA1c[d] at baseline, %	HbA1c at f/u visit, %	HbA1c difference, %	*P*	Adjustment model[e], 95% CI		
		physician	nursing	nutritional					HbA1c difference, (%)	f/u HbA1c, (%)	*P*
1st	29	4.3±1.0	3.2±1.0	3.2±1.0	8.56±1.93	7.46±1.16	1.51±1.87	.01	1.53 (0.82 - 2.24)	7.46 (7.25 - 7.67)	.07
2nd	27	4.4±1.1	3.2±1.0	3.2±1.0	8.44±1.98	7.59±1.30	1.55±1.79	.05	1.57 (0.84 - 2.30)	7.59 (7.40 - 7.79)	.38
3rd	24	4.3±1.1	3.4±0.9	3.4±0.8	8.68±1.93	7.60±1.21	1.58±1.80	.02	1.63 (0.79 - 2.46)	7.59 (7.37 - 7.81)	.15
4th	22	3.9±0.6	3.2±1.1	3.2±0.9	8.31±1.61	7.66±1.40	1.34±1.40	.11	1.32 (0.65 - 2.00)	7.67 (7.43 - 7.91)	.77
5th	20	4.0±0.0	3.3±1.0	3.4±0.8	8.28±1.48	7.70±1.34	1.43±1.45	.21	1.44 (0.65 - 2.23)	7.70 (7.49 - 7.91)	> .99

[a] Home and Self-care Program (HELP) means receiving at least one structured education session (with remote digital support) comprising nursing and nutrition education and a physician consultation.

[b] T1D: type 1 diabetes.

[c] Follow-up visit after the corresponding number of structured educations session(s).

[d] HbA1c: hemoglobin A1c

[e] Adjusted for age, sex, , body mass index, HbA1c, treatment type, and diabetes duration.

Table S4. Effects of HELP[a] on glycemic control in T1D[b] patients with SES[c] information (n = 63).

No. of education	f/u visit[d]	Subjects	HbA1c[e] (%) at baseline	HbA1c[e] (%) at f/u visit	HbA1c difference, %	*P*	Adjustment model, 95% CI: difference (Model 1[f])	Adjustment model, 95% CI: difference (Model 2[g])	Adjustment model, 95% CI: f/u HbA1c (Model 2)
≥ 1	1st	56	8.66 ± 2.26	7.4 ± 1.19	1.54 ± 2.19	.002	1.62 (1.00 - 2.24)	1.75 (1.14 - 2.36)	7.39 (7.22 - 7.56)
	2nd	52	8.59 ± 2.10	7.42 ± 1.17	1.60 ± 2.00	.004	1.65 (1.06 - 2.24)	1.78 (1.21 - 2.35)	7.43 (7.22 - 7.64)
	3rd	54	8.60 ± 2.11	7.42 ± 1.38	1.57 ± 2.02	.003	1.64 (1.05 - 2.23)	1.80 (1.24 - 2.36)	7.43 (7.19 - 7.67)
	4th	53	8.64 ± 2.11	7.32 ± 1.00	1.57 ± 2.04	.001	1.62 (1.01 - 2.23)	1.80 (1.24 - 2.36)	7.34 (7.15 - 7.52)
	5th	47	8.66 ± 2.16	7.58 ± 1.10	1.57 ± 2.04	.02	1.67 (1.00 - 2.33)	1.85 (1.23 - 2.48)	7.55 (7.36 - 7.74)
	6th	39	8.77 ± 2.18	7.68 ± 1.13	1.54 ± 1.94	.04	1.57 (0.89 - 2.25)	1.74 (1.10 - 2.38)	7.65 (7.43 - 7.87)
	7th	38	8.60 ± 1.99	7.56 ± 1.17	1.41 ± 1.66	.02	1.42 (0.81 - 2.03)	1.57 (0.92 - 2.21)	7.52 (7.26 - 7.77)
	8th	33	8.48 ± 1.35	7.54 ± 1.18	1.23 ± 1.31	.01	1.28 (0.78 - 1.77)	1.32 (0.80 - 1.85)	7.56 (7.37 - 7.74)
≥ 2	1st	46	8.82 ± 2.10	7.52 ± 1.25	1.6 ± 2.06	.002	1.61 (0.94 - 2.28)	1.85 (1.20 - 2.50)	7.53 (7.32 - 7.75)
	2nd	46	8.82 ± 2.10	7.5 ± 1.25	1.67 ± 2.05	.002	1.67 (1.00 - 2.34)	1.91 (1.27 - 2.54)	7.54 (7.27 - 7.81)
	3rd	45	8.85 ± 2.11	7.43 ± 1.02	1.62 ± 2.08	.001	1.64 (0.96 - 2.33)	1.97 (1.35 - 2.59)	7.41 (7.17 - 7.65)
	4th	41	8.92 ± 2.15	7.69 ± 1.09	1.61 ± 2.17	.02	1.69 (0.92 - 2.46)	1.99 (1.26 - 2.73)	7.65 (7.42 - 7.88)
	5th	36	8.89 ± 2.20	7.74 ± 1.14	1.56 ± 2.02	.03	1.59 (0.86 - 2.32)	1.76 (1.10 - 2.43)	7.71 (7.48 - 7.93)
	6th	34	8.57 ± 1.99	7.49 ± 1.21	1.51 ± 1.70	.03	1.49 (0.85 - 2.14)	1.61 (0.93 - 2.28)	7.49 (7.24 - 7.74)
	7th	29	8.42 ± 1.29	7.45 ± 1.14	1.25 ± 1.37	.02	1.27 (0.72 - 1.82)	1.30 (0.72 - 1.87)	7.45 (7.29 - 7.61)
≥ 3	1st	38	8.96 ± 2.19	7.59 ± 1.27	1.68 ± 2.15	.006	1.68 (0.92 - 2.44)	1.85 (1.15 - 2.54)	7.62 (7.32 - 7.92)
	2nd	38	8.96 ± 2.19	7.48 ± 0.97	1.62 ± 2.16	.001	1.64 (0.88 - 2.40)	1.84 (1.16 - 2.52)	7.45 (7.19 - 7.71)
	3rd	35	9.11 ± 2.22	7.67 ± 1.06	1.71 ± 2.31	.01	1.75 (0.89 - 2.61)	1.96 (1.15 - 2.76)	7.66 (7.41 - 7.91)
	4th	30	9.11 ± 2.30	7.71 ± 1.02	1.61 ± 2.18	.02	1.60 (0.79 - 2.41)	1.67 (1.00 - 2.35)	7.68 (7.46 - 7.90)
	5th	29	8.78 ± 2.08	7.52 ± 1.11	1.54 ± 1.78	.01	1.53 (0.80 - 2.25)	1.56 (0.83 - 2.30)	7.51 (7.25 - 7.77)
	6th	25	8.59 ± 1.29	7.43 ± 1.05	1.25 ± 1.41	.004	1.24 (0.64 - 1.83)	1.22 (0.55 - 1.89)	7.43 (7.24 - 7.62)
≥ 4	1st	26	8.99 ± 2.32	7.34 ± 0.93	1.77 ± 2.5	.02	1.76 (0.68 - 2.84)	1.85 (0.89 - 2.81)	7.34 (7.05 - 7.62)
	2nd	26	8.99 ± 2.32	7.63 ± 1.12	1.64 ± 2.32	.05	1.65 (0.68 - 2.61)	1.71 (0.77 - 2.66)	7.63 (7.33 - 7.93)
	3rd	25	9.02 ± 2.35	7.5 ± 1.03	1.62 ± 2.28	.02	1.63 (0.65 - 2.61)	1.58 (0.65 - 2.50)	7.50 (7.26 - 7.74)
	4th	23	8.42 ± 1.08	7.37 ± 1.01	1.18 ± 1.30	.009	1.18 (0.61 - 1.74)	1.16 (0.50 - 1.82)	7.38 (7.15 - 7.62)
	5th	21	8.37 ± 1.12	7.43 ± 0.98	1.21 ± 1.41	.07	1.20 (0.60 - 1.81)	1.21 (0.45 - 1.97)	7.42 (7.15 - 7.70)

Values are expressed as n (%), mean ± standard deviation, percentage, or median (interquartile range).

[a] Home and Self-care Program (HELP) means receiving at least one structured education session (with remote digital support) comprising nursing and nutrition education and a physician consultation.

[b] T1D: type 1 diabetes.

[c] SES: socioeconomic status

[d] Follow-up visit after the corresponding number of structured educations session(s).

[e] HbA1c: hemoglobin A1c

[f] Model 1: adjusted for age, sex, body mass index, and HbA1c.

[g] Model 2: Model 1 + additional adjustment for treatment type, diabetes duration., schooling, social status, and employment status.

Table S5. Effects of HELP[a] on glycemic control in CGM[b] users (n = 99).

No. of education	f/u visit[c]	Subjects	HbA1c[d] (%) at baseline	HbA1c[d] (%) at f/u visit	HbA1c difference, %	*P*	HbA1c difference, 95% CI Model 1[e]	HbA1c difference, 95% CI Model 2[f]
≥ 1	1st	69	8.60 ± 2.29	7.31 ± 1.12	1.69 ± 2.15	< .001	1.74 (1.21 - 2.27)	1.75 (1.23 - 2.27)
	2nd	60	8.63 ± 2.19	7.38 ± 1.22	1.77 ± 2.06	.001	1.82 (1.27 - 2.36)	1.83 (1.29 - 2.37)
	3rd	58	8.71 ± 2.24	7.52 ± 1.39	1.72 ± 2.15	.005	1.75 (1.16 - 2.33)	1.77 (1.20 - 2.35)
	4th	49	8.70 ± 2.20	7.45 ± 1.25	1.80 ± 2.21	.01	1.82 (1.14 - 2.50)	1.85 (1.17 - 2.52)
	5th	43	8.69 ± 2.25	7.56 ± 1.11	1.67 ± 2.10	.03	1.74 (1.03 - 2.44)	1.77 (1.07 - 2.47)
	6th	35	8.83 ± 2.28	7.62 ± 1.12	1.67 ± 2.00	.03	1.67 (0.94 - 2.40)	1.69 (0.95 - 2.43)
	7th	32	8.68 ± 2.14	7.58 ± 1.17	1.47 ± 1.78	.04	1.47 (0.77 - 2.18)	1.48 (0.75 - 2.21)
	8th	28	8.54 ± 1.43	7.60 ± 1.2	1.29 ± 1.37	.04	1.31 (0.74 - 1.88)	1.32 (0.73 - 1.90)
≥ 2	1st	43	8.97 ± 2.14	7.53 ± 1.24	1.75 ± 2.11	.002	1.76 (1.07 - 2.45)	1.81 (1.12 - 2.50)
	2nd	42	8.96 ± 2.16	7.66 ± 1.24	1.68 ± 2.15	.008	1.65 (0.93 - 2.38)	1.72 (1.00 - 2.44)
	3rd	41	8.97 ± 2.19	7.47 ± 1.06	1.71 ± 2.15	.001	1.73 (1.00 - 2.45)	1.79 (1.06 - 2.51)
	4th	38	9.01 ± 2.21	7.66 ± 1.11	1.77 ± 2.20	.01	1.81 (1.02 - 2.61)	1.87 (1.07 - 2.67)
	5th	32	8.97 ± 2.31	7.66 ± 1.15	1.69 ± 2.10	.03	1.70 (0.90 – 2.50)	1.73 (0.93 - 2.54)
	6th	28	8.65 ± 2.17	7.51 ± 1.20	1.61 ± 1.84	.07	1.59 (0.82 - 2.36)	1.59 (0.80 - 2.39)
	7th	24	8.47 ± 1.38	7.50 ± 1.17	1.31 ± 1.45	.07	1.31 (0.65 - 1.97)	1.31 (0.62 - 2.00)
≥ 3	1st	34	9.09 ± 2.27	7.78 ± 1.24	1.66 ± 2.28	.03	1.63 (0.78 - 2.48)	1.69 (0.84 - 2.54)
	2nd	33	9.10 ± 2.30	7.52 ± 0.95	1.74 ± 2.27	.003	1.75 (0.89 - 2.61)	1.80 (0.94 - 2.65)
	3rd	31	9.23 ± 2.32	7.71 ± 1.04	1.83 ± 2.41	.02	1.84 (0.89 - 2.80)	1.90 (0.94 - 2.86)
	4th	26	9.25 ± 2.43	7.73 ± 1.00	1.75 ± 2.29	.02	1.75 (0.84 - 2.66)	1.75 (0.85 - 2.66)
	5th	24	8.89 ± 2.25	7.55 ± 1.04	1.62 ± 1.93	.03	1.62 (0.73 - 2.50)	1.62 (0.69 - 2.55)
	6th	21	8.66 ± 1.36	7.48 ± 1.04	1.29 ± 1.48	.02	1.25 (0.52 - 1.97)	1.23 (0.47 - 2.00)
≥ 4	1st	23	9.11 ± 2.43	7.37 ± 0.87	1.87 ± 2.62	.03	1.87 (0.65 - 3.09)	1.86 (0.62 - 3.09)
	2nd	23	9.11 ± 2.43	7.70 ± 1.09	1.73 ± 2.43	.09	1.72 (0.63 - 2.82)	1.72 (0.56 - 2.87)
	3rd	22	9.14 ± 2.47	7.52 ± 0.99	1.73 ± 2.40	.03	1.80 (0.66 - 2.94)	1.75 (0.56 - 2.95)
	4th	19	8.46 ± 1.14	7.37 ± 0.86	1.16 ± 1.39	.02	1.15 (0.45 - 1.85)	1.14 (0.38 - 1.91)
	5th	17	8.41 ± 1.20	7.49 ± 0.95	1.25 ± 1.51	.23	1.23 (0.50 - 1.96)	1.22 (0.41 - 2.03)

Values are expressed as n (%), mean ± standard deviation, percentage, or median (interquartile range).

[a] Home and Self-care Program (HELP) means receiving at least one structured education session (with remote digital support) comprising nursing and nutrition education and a physician consultation.

[b] CGM: continuous glucose monitoring.

[c] Follow-up visit after the corresponding number of structured educations session(s).

[d] HbA1c: hemoglobin A1c

[e] Model 1: adjusted for age, sex, , body mass index, and HbA1c.

[f] Model 2: Model 1 + additional adjustment for treatment type, and diabetes duration.

Table S6. Effects of HELP[a] on glycemic control in CGM[b] users with SES[c] information (n = 53).

No. of education	f/u visit[d]	Subjects	HbA1c[e] (%) at baseline	HbA1c[e] (%) at f/u visit	HbA1c difference, %	*P*	HbA1c difference, 95% CI Model 1[f]	HbA1c difference, 95% CI Model 2[g]
≥ 1	1st	48	8.77 ± 2.41	7.43 ± 1.20	1.63 ± 2.34	.005	1.71 (0.99 - 2.42)	1.84 (1.13 - 2.55)
	2nd	44	8.70 ± 2.25	7.49 ± 1.24	1.73 ± 2.13	.02	1.78 (1.10 - 2.46)	1.93 (1.27 - 2.59)
	3rd	46	8.70 ± 2.26	7.52 ± 1.41	1.66 ± 2.16	.02	1.70 (1.02 - 2.39)	1.87 (1.22 - 2.51)
	4th	45	8.76 ± 2.25	7.36 ± 1.00	1.69 ± 2.18	.002	1.74 (1.03 - 2.44)	1.93 (1.28 - 2.57)
	5th	41	8.72 ± 2.30	7.60 ± 1.11	1.68 ± 2.15	.05	1.76 (1.01 - 2.51)	1.94 (1.24 - 2.64)
	6th	33	8.87 ± 2.34	7.67 ± 1.11	1.68 ± 2.06	.06	1.70 (0.91 - 2.48)	1.86 (1.12 - 2.61)
	7th	32	8.68 ± 2.14	7.58 ± 1.17	1.47 ± 1.78	.04	1.47 (0.77 - 2.18)	1.61 (0.83 - 2.38)
	8th	28	8.54 ± 1.43	7.60 ± 1.20	1.29 ± 1.37	.04	1.31 (0.74 - 1.88)	1.37 (0.75 – 2.00)
≥ 2	1st	38	8.99 ± 2.26	7.59 ± 1.28	1.76 ± 2.21	.008	1.77 (0.97 - 2.57)	2.06 (1.29 - 2.82)
	2nd	38	8.99 ± 2.26	7.66 ± 1.28	1.75 ± 2.24	.02	1.73 (0.92 - 2.54)	2.02 (1.26 - 2.79)
	3rd	38	8.99 ± 2.26	7.47 ± 1.03	1.76 ± 2.22	.002	1.78 (0.98 - 2.58)	2.11 (1.39 - 2.83)
	4th	35	9.04 ± 2.29	7.72 ± 1.11	1.77 ± 2.29	.03	1.83 (0.95 - 2.70)	2.10 (1.25 - 2.95)
	5th	30	9.03 ± 2.38	7.72 ± 1.14	1.72 ± 2.16	.05	1.73 (0.87 - 2.59)	1.88 (1.11 - 2.65)
	6th	28	8.65 ± 2.17	7.51 ± 1.20	1.61 ± 1.84	.07	1.59 (0.82 - 2.36)	1.67 (0.84 - 2.51)
	7th	24	8.47 ± 1.38	7.50 ± 1.17	1.31 ± 1.45	.07	1.31 (0.65 - 1.97)	1.36 (0.63 - 2.09)
≥ 3	1st	32	9.12 ± 2.34	7.75 ± 1.28	1.74 ± 2.33	.03	1.72 (0.81 - 2.63)	1.90 (1.07 - 2.74)
	2nd	32	9.12 ± 2.34	7.52 ± 0.97	1.76 ± 2.3	.004	1.77 (0.88 - 2.67)	1.97 (1.18 - 2.76)
	3rd	30	9.25 ± 2.36	7.72 ± 1.06	1.86 ± 2.45	.02	1.88 (0.88 - 2.88)	2.07 (1.14 – 3.00)
	4th	25	9.28 ± 2.47	7.73 ± 1.02	1.79 ± 2.33	.03	1.78 (0.83 - 2.73)	1.75 (0.95 - 2.55)
	5th	24	8.89 ± 2.25	7.55 ± 1.04	1.62 ± 1.93	.03	1.62 (0.73 - 2.50)	1.59 (0.65 - 2.52)
	6th	21	8.66 ± 1.36	7.48 ± 1.04	1.29 ± 1.48	.02	1.25 (0.52 - 1.97)	1.26 (0.39 - 2.13)
≥ 4	1st	22	9.13 ± 2.48	7.37 ± 0.89	1.90 ± 2.67	.04	1.90 (0.61 - 3.19)	1.89 (0.71 - 3.06)
	2nd	22	9.13 ± 2.48	7.70 ± 1.11	1.75 ± 2.49	.10	1.75 (0.60 - 2.91)	1.77 (0.58 - 2.95)
	3rd	21	9.17 ± 2.52	7.51 ± 1.01	1.77 ± 2.45	.04	1.82 (0.62 - 3.01)	1.59 (0.36 - 2.82)
	4th	19	8.46 ± 1.14	7.37 ± 0.86	1.16 ± 1.39	.02	1.15 (0.45 - 1.85)	1.15 (0.25 - 2.05)
	5th	17	8.41 ± 1.20	7.49 ± 0.95	1.25 ± 1.51	.23	1.23 (0.50 - 1.96)	1.31 (0.30 - 2.31)

Values are expressed as n (%), mean ± standard deviation, percentage, or median (interquartile range).
[a] Home and Self-care Program (HELP) means receiving at least one structured education session (with remote digital support) comprising nursing and nutrition education and a physician consultation.
[b] CGM: continuous glucose monitoring.
[c] SES: socioeconomic status
[d] Follow-up period visit the corresponding number of structured educations session(s).
[e] HbA1c: hemoglobin A1c
[f] Model 1: adjusted for age, sex, body mass index, and HbA1c.
[g] Model 2: Model 1 + additional adjustment for treatment type, diabetes duration., schooling, social status, and employment status.

Table S7. Effects of HELP[a] on glycemic control in CGM[b] users with '% time CGM active' of ≥70%.

No. of education	f/u visit[c]	Subjects	HbA1c[d] (%) at baseline	HbA1c[d] (%) at f/u visit	HbA1c difference, %	*P*	HbA1c difference, 95% CI Model 1[e]	HbA1c difference, 95% CI Model 2[f]
≥ 1	1st	33	8.65 ± 2.06	7.01 ± 1.02	2.02 ± 2.08	.003	2.05 (1.33 - 2.77)	2.05 (1.32 - 2.79)
	2nd	36	8.74 ± 2.05	7.03 ± 0.99	1.91 ± 2.06	< .001	2.11 (1.40 - 2.82)	2.16 (1.45 - 2.86)
	3rd	31	9.12 ± 2.03	7.24 ± 1.20	2.05 ± 2.14	.001	2.08 (1.35 - 2.81)	2.12 (1.40 - 2.85)
	4th	27	9.09 ± 1.77	7.46 ± 1.05	1.73 ± 2.07	.005	1.90 (1.08 - 2.73)	2.83 (1.22 - 4.43)
	5th	27	8.89 ± 1.79	7.40 ± 0.95	1.71 ± 1.87	.007	1.90 (1.19 - 2.62)	2.44 (1.11 - 3.76)
	6th	25	8.73 ± 2.26	7.38 ± 0.98	1.67 ± 1.91	.04	1.64 (0.81 - 2.48)	1.66 (0.80 - 2.52)
	7th	23	9.06 ± 2.33	7.56 ± 1.14	1.70 ± 1.96	.02	1.70 (0.70 - 2.70)	1.73 (0.66 - 2.80)
	8th	18	8.66 ± 1.38	7.26 ± 1.11	1.43 ± 1.57	.01	1.46 (0.62 - 2.30)	1.47 (0.55 - 2.39)
≥ 2	1st	24	9.06 ± 1.84	7.10 ± 1.02	2.12 ± 2.09	.002	2.21 (1.30 - 3.12)	2.30 (1.40 - 3.19)
	2nd	26	9.17 ± 1.81	7.48 ± 1.24	1.90 ± 2.03	.005	1.91 (1.12 - 2.70)	1.97 (1.19 - 2.75)
	3rd	25	9.32 ± 1.81	7.48 ± 1.15	1.96 ± 2.07	.002	2.01 (1.16 - 2.87)	2.08 (1.24 - 2.93)
	4th	24	8.92 ± 1.87	7.33 ± 0.93	1.76 ± 1.96	.009	1.89 (1.05 - 2.73)	2.51 (0.97 - 4.05)
	5th	22	8.97 ± 2.28	7.46 ± 1.00	1.74 ± 2.05	.03	1.71 (0.75 - 2.67)	2.13 (0.41 - 3.85)
	6th	20	9.05 ± 2.43	7.39 ± 1.20	1.90 ± 2.01	.02	1.87 (0.79 - 2.94)	1.88 (0.72 - 3.05)
	7th	16	8.63 ± 1.26	7.21 ± 1.03	1.45 ± 1.63	.03	1.45 (0.63 - 2.27)	1.45 (0.56 - 2.34)
≥ 3	1st	22	9.30 ± 1.84	7.68 ± 1.23	1.86 ± 2.06	.02	1.84 (0.98 - 2.70)	1.87 (0.99 - 2.74)
	2nd	22	9.40 ± 1.82	7.64 ± 1.12	1.89 ± 2.06	.006	1.91 (0.99 - 2.82)	1.94 (1.02 - 2.86)
	3rd	20	9.23 ± 1.87	7.50 ± 0.90	1.91 ± 2.11	.02	1.95 (0.99 - 2.91)	2.63 (0.82 - 4.44)
	4th	18	9.34 ± 2.36	7.73 ± 0.91	1.89 ± 2.22	.07	1.83 (0.76 - 2.90)	1.95 (-0.18 - 4.09)
	5th	17	9.38 ± 2.49	7.60 ± 1.18	2.05 ± 2.13	.04	2.02 (0.78 - 3.26)	2.03 (0.66 - 3.40)
	6th	14	8.85 ± 1.16	7.33 ± 1.04	1.55 ± 1.71	.04	1.53 (0.54 - 2.52)	1.50 (0.40 - 2.61)
≥ 4	1st	16	9.04 ± 1.79	7.46 ± 1.02	1.76 ± 2.15	.07	1.77 (0.64 - 2.89)	1.75 (0.57 - 2.93)
	2nd	15	9.71 ± 2.76	7.55 ± 0.96	2.29 ± 2.85	.07	2.30 (0.63 - 3.96)	2.44 (-0.67 - 5.55)
	3rd	14	9.47 ± 2.46	7.52 ± 0.90	1.98 ± 2.38	.05	2.08 (0.45 - 3.71)	2.59 (-0.31 - 5.49)
	4th	15	8.75 ± 1.09	7.41 ± 0.94	1.38 ± 1.49	.02	1.38 (0.52 - 2.23)	1.37 (0.41 - 2.34)
	5th	12	8.59 ± 1.28	7.32 ± 1.07	1.49 ± 1.72	.20	1.23 (0.19 - 2.27)	1.22 (-0.13 - 2.56)

Values are expressed as n (%), mean ± standard deviation, percentage, or median (interquartile range).

[a] Home and Self-care Program (HELP) means receiving at least one structured education session (with remote digital support) comprising nursing and nutrition education and a physician consultation.

[b] CGM: continuous glucose monitoring.

[c] Follow-up visit after the corresponding number of structured educations session(s).

[d] HbA1c: hemoglobin A1c

[e] Model 1: adjusted for age, sex, body mass index, and HbA1c.

[f] Model 2: Model 1 + additional adjustment for treatment type, and diabetes duration.

Table S8. Effects of HELP[a] on CGM[b] metrics in patients with T1D[c] (n = 99).

No. of education	f/u visit[d]	Subjects	AGP[e] (%)							
			at f/u visit				Adjustment model[i], 95% CI			
			TBR[f] (70)	TBR (54)	TIR[g]	CV[h]	TBR (<70 mg/dL)	TBR (<54 mg/dL)	TIR	CV
≥1	1st	69	6.82 ± 8.11	1.76 ± 3.48	61.62 ± 17.66	39.48 ± 8.45	6.85 (4.72 - 8.98)	1.77 (0.78 - 2.77)	61.41 (58.21 - 64.61)	39.38 (37.07 - 41.68)
	2nd	60	6.06 ± 6.53	1.91 ± 3.49	61.77 ± 16.79	40.72 ± 7.74	6.07 (4.24 - 7.91)	2.01 (1.00 - 3.02)	61.59 (58.14 - 65.03)	40.55 (38.41 - 42.69)
	3rd	58	6.47 ± 8.71	2.09 ± 4.52	57.45 ± 17.13	40.17 ± 7.98	6.49 (4.15 - 8.83)	2.08 (0.77 - 3.39)	57.29 (53.55 - 61.03)	39.86 (37.55 - 42.18)
	4th	49	6.01 ± 7.12	1.50 ± 3.39	57.63 ± 16.40	41.86 ± 15.74	6.52 (4.44 - 8.60)	1.78 (0.72 - 2.85)	57.12 (53.52 - 60.71)	41.12 (36.79 - 45.45)
	5th	43	6.37 ± 9.78	1.38 ± 3.67	56.51 ± 18.11	39.25 ± 8.08	6.54 (3.28 - 9.81)	1.78 (0.56 – 3.00)	56.01 (51.32 - 60.70)	39.68 (36.99 - 42.38)
	6th	35	3.97 ± 4.15	0.71 ± 1.40	57.12 ± 16.53	40.01 ± 7.69	3.86 (2.39 - 5.33)	0.67 (0.13 - 1.21)	56.3 0(53.04 - 59.55)	39.73 (36.88 - 42.57)
	7th	32	3.84 ± 4.31	0.55 ± 1.09	56.61 ± 18.70	38.44 ± 5.67	3.39 (2.10 - 4.68)	0.39 (0.01 - 0.77)	55.99 (51.96 - 60.03)	38.10 (35.94 - 40.27)
	8th	28	3.43 ± 4.37	0.44 ± 0.89	55.85 ± 20.61	37.15 ± 5.60	3.16 (1.68 - 4.64)	0.36 (0.04 - 0.69)	54.70 (50.92 - 58.48)	36.77 (34.35 - 39.18)
P-trend			.003	.001	.004[j]	.25[j]	.006	.005	.01[j]	.13[j]
≥2	1st	43	4.44 ± 3.42	1.22 ± 1.97	58.51 ± 17.60	39.77 ± 6.38	4.51 (3.38 - 5.64)	1.29 (0.63 - 1.94)	58.56 (54.3 - 62.82)	39.91 (37.51 - 42.31)
	2nd	42	4.92 ± 5.54	1.45 ± 2.29	55.47 ± 17.04	39.68 ± 6.69	4.89 (3.06 - 6.72)	1.43 (0.61 - 2.26)	55.33 (51.41 - 59.25)	39.78 (37.28 - 42.28)
	3rd	41	5.09 ± 4.36	1.08 ± 1.57	59.03 ± 17.80	42.13 ± 16.85	5.14 (3.84 - 6.43)	1.05 (0.53 - 1.57)	57.82 (54.44 - 61.20)	40.53 (35.73 - 45.32)
	4th	38	4.84 ± 4.54	0.89 ± 1.71	57.70 ± 18.10	40.86 ± 7.58	4.74 (3.13 - 6.36)	0.91 (0.28 - 1.55)	56.19 (51.72 - 60.66)	40.30 (37.54 - 43.06)
	5th	32	3.81 ± 4.13	0.74 ± 1.48	57.94 ± 16.84	39.44 ± 6.06	3.67 (2.17 - 5.17)	0.69 (0.08 - 1.30)	57.10 (53.79 - 60.42)	39.27 (36.97 - 41.56)
	6th	28	3.25 ± 3.58	0.48 ± 0.98	56.81 ± 19.54	37.79 ± 5.40	3.08 (1.80 - 4.36)	0.39 (0.02 - 0.76)	56.49 (52.48 - 60.49)	37.69 (35.50 - 39.88)
	7th	24	3.67 ± 4.60	0.48 ± 1.08	55.91 ± 21.58	36.51 ± 5.97	3.67 (1.92 - 5.41)	0.46 (0.03 - 0.89)	55.66 (51.90 - 59.41)	36.46 (33.87 - 39.05)
P-trend			.05	.002	.002[j]	.23[j]	.05	.001	.004[j]	.09[j]
≥3	1st	34	4.72 ± 4.76	1.43 ± 2.21	55.03 ± 17.03	40.73 ± 6.90	4.66 (2.99 - 6.32)	1.42 (0.56 - 2.28)	54.86 (50.37 - 59.35)	40.49 (37.73 - 43.25)
	2nd	33	5.41 ± 4.48	1.23 ± 1.7	57.37 ± 15.82	40.12 ± 6.64	5.32 (4.01 - 6.64)	1.14 (0.59 - 1.69)	57.07 (53.48 - 60.65)	39.72 (37.12 - 42.31)
	3rd	31	5.16 ± 4.86	1.07 ± 1.86	55.80 ± 16.75	40.66 ± 6.89	5.06 (3.27 - 6.86)	1.05 (0.34 - 1.77)	55.3 (50.19 - 60.41)	40.42 (37.80 - 43.04)
	4th	26	3.65 ± 4.34	0.80 ± 1.55	55.28 ± 15.28	39.76 ± 6.32	3.65 (2.02 - 5.29)	0.78 (0.12 - 1.44)	55.17 (51.32 - 59.02)	39.66 (37.26 - 42.07)
	5th	24	3.04 ± 3.37	0.43 ± 0.90	55.96 ± 17.44	38.38 ± 5.26	3.04 (1.69 - 4.39)	0.42 (0.04 - 0.80)	55.86 (51.14 - 60.58)	38.33 (35.98 - 40.68)
	6th	21	3.90 ± 4.85	0.55 ± 1.15	55.20 ± 19.01	37.41 ± 5.88	4.23 (2.27 - 6.19)	0.61 (0.12 - 1.11)	55.80 (51.34 - 60.27)	37.69 (34.96 - 40.41)
P-trend			.10	.002	.04[j]	.21[j]	.02	.005	.04[j]	.12[j]
≥4	1st	23	5.59 ± 4.37	1.40 ± 1.60	59.00 ± 13.72	40.42 ± 7.24	5.64 (3.99 - 7.29)	1.40 (0.76 - 2.04)	59.00 (53.78 - 64.22)	40.42 (37.27 - 43.56)
	2nd	23	5.70 ± 5.11	1.36 ± 2.04	57.05 ± 13.64	40.84 ± 7.28	5.75 (3.92 - 7.58)	1.36 (0.52 - 2.21)	57.05 (52.53 - 61.56)	40.84 (37.70 - 43.98)

3rd	22	4.32 ± 4.77	0.95 ± 1.69	55.86 ± 14.91	40.07 ± 7.32	4.90 (2.98 - 6.82)	1.11 (0.41 - 1.82)	55.92 (51.13 - 60.72)	40.66 (37.68 - 43.63)
4th	19	3.84 ± 3.79	0.67 ± 1.19	58.83 ± 15.52	39.68 ± 5.55	4.20 (2.50 - 5.91)	0.74 (0.17 - 1.32)	59.24 (53.11 - 65.37)	39.92 (36.99 - 42.86)
5th	17	4.12 ± 4.96	0.44 ± 0.89	57.50 ± 16.40	37.91 ± 5.24	4.58 (2.52 - 6.65)	0.50 (0.04 - 0.96)	57.88 (52.15 - 63.61)	38.00 (34.83 - 41.17)
P-trend		.05	.003	.005[j]	.35[j]	.06	.005	.005[j]	.48[j]

[a] Home and Self-care Program (HELP) means receiving at least one structured education session (with remote digital support) comprising nursing and nutrition education and a physician consultation.

[b] CGM: continuous glucose monitoring.

[c] T1D: type 1 diabetes.

[d] Follow-up visit after the corresponding number of structured educations session(s).

[e] AGP: ambulatory glucose profile.

[f] TBR: time below range

[g] TIR: time in target range

[h] CV: coefficient of variation

[I] Adjusted for age, sex, body mass index, hemoglobin A1c, treatment type, and diabetes duration.

[j] *P*-value (in t-test) for maintenance above reference (TIR 55.0%) and below reference (CV 40.0%).

Table S9. Effects of HELP[a] on CGM[b] metrics in T1D[c] patients with SES[d] information (n = 53).

No. of education	f/u visit[e]	Subjects	AGP[f] (%) at f/u visit TBR[g] (70)	TBR (54)	TIR[h]	CV[i]	Adjustment model[j], 95% CI TBR (<70 mg/dL)	TBR (<54 mg/dL)	TIR	CV
≥1	1st	48	8.14 ± 8.50	2.44 ± 4.14	59.75 ± 16.55	41.26 ± 8.37	7.87 (4.97 - 10.78)	2.36 (0.52 - 4.19)	59.78 (55.53 - 64.02)	40.95 (37.30 - 44.61)
	2nd	44	6.89 ± 6.93	2.46 ± 3.99	60.43 ± 15.81	41.55 ± 7.18	7.16 (4.67 - 9.64)	2.66 (1.07 - 4.25)	60.47 (56.25 - 64.68)	41.00 (38.17 - 43.83)
	3rd	46	7.40 ± 9.40	2.60 ± 5.01	56.96 ± 16.27	40.83 ± 7.35	7.39 (4.6 - 10.17)	2.64 (0.89 - 4.40)	56.37 (52.55 - 60.18)	40.35 (37.62 - 43.08)
	4th	45	6.37 ± 7.29	1.62 ± 3.53	58.09 ± 15.85	39.66 ± 6.99	7.16 (4.94 - 9.38)	2.13 (0.87 - 3.39)	57.22 (53.53 - 60.91)	39.67 (37.06 - 42.28)
	5th	41	6.56 ± 9.97	1.45 ± 3.75	55.83 ± 17.94	39.45 ± 8.23	6.55 (2.99 - 10.11)	2.06 (0.70 - 3.42)	55.61 (50.51 - 60.71)	40.05 (37.02 - 43.08)
	6th	33	4.06 ± 4.25	0.75 ± 1.44	56.31 ± 16.22	40.30 ± 7.83	4.00 (2.39 - 5.61)	0.81 (0.24 - 1.38)	55.39 (51.70 - 59.08)	39.95 (36.72 - 43.18)
	7th	32	3.84 ± 4.31	0.55 ± 1.09	56.61 ± 18.70	38.44 ± 5.67	3.36 (1.96 - 4.76)	0.41 (0.00 - 0.84)	56.41 (52.31 - 60.52)	37.94 (35.53 - 40.34)
	8th	28	3.43 ± 4.37	0.44 ± 0.89	55.85 ± 20.61	37.15 ± 5.60	3.29 (1.75 - 4.82)	0.39 (0.03 - 0.75)	55.00 (51.01 - 58.99)	36.67 (33.96 - 39.39)
P-trend			< .001	< .001	.003[k]	.38[k]	.001	.002	.01[k]	.23[k]
≥2	1st	38	4.85 ± 3.41	1.42 ± 2.06	58.46 ± 18.18	40.18 ± 6.70	4.75 (3.45 - 6.06)	1.23 (0.39 - 2.07)	59.6 (54.05 - 65.16)	39.33 (36.15 - 42.51)
	2nd	38	5.31 ± 5.69	1.62 ± 2.36	56.24 ± 17.58	40.15 ± 6.91	4.69 (2.62 - 6.77)	1.26 (0.23 - 2.29)	56.39 (51.70 - 61.08)	39.07 (36.01 - 42.13)
	3rd	38	5.41 ± 4.37	1.17 ± 1.60	59.43 ± 16.87	39.81 ± 6.47	5.40 (3.98 - 6.82)	1.05 (0.47 - 1.63)	58.46 (54.47 - 62.44)	38.90 (36.20 - 41.59)
	4th	35	5.11 ± 4.61	0.97 ± 1.77	57.59 ± 17.88	41.24 ± 7.78	5.03 (3.21 - 6.86)	1.07 (0.36 - 1.77)	55.76 (50.73 - 60.80)	40.55 (37.26 - 43.85)
	5th	30	3.90 ± 4.24	0.79 ± 1.52	57.10 ± 16.57	39.73 ± 6.17	3.88 (2.29 - 5.47)	0.84 (0.19 - 1.49)	56.13 (52.36 - 59.91)	39.36 (36.83 - 41.88)
	6th	28	3.25 ± 3.58	0.48 ± 0.98	56.81 ± 19.54	37.79 ± 5.40	3.11 (1.75 - 4.48)	0.42 (0.01 - 0.83)	56.64 (52.30 - 60.99)	37.50 (35.07 - 39.92)
	7th	24	3.67 ± 4.60	0.48 ± 1.08	55.91 ± 21.58	36.51 ± 5.97	3.75 (1.95 - 5.56)	0.45 (0.00 - 0.91)	56.01 (52.07 - 59.96)	36.30 (33.50 - 39.11)
P-trend			.03	.001	.001[k]	.16[k]	.06	.002	.005[k]	.03[k]
≥3	1st	32	4.99 ± 4.79	1.54 ± 2.25	55.18 ± 17.63	41.08 ± 7.02	4.70 (2.83 - 6.58)	1.22 (0.22 - 2.23)	55.75 (50.32 - 61.19)	39.61 (36.53 - 42.69)
	2nd	32	5.55 ± 4.48	1.28 ± 1.71	57.48 ± 16.08	40.26 ± 6.71	5.54 (4.13 - 6.95)	1.15 (0.52 - 1.77)	57.23 (53.40 - 61.07)	39.20 (36.36 - 42.05)
	3rd	30	5.30 ± 4.88	1.10 ± 1.88	55.86 ± 17.04	40.82 ± 6.95	5.09 (3.18 - 7.01)	1.11 (0.37 - 1.86)	54.95 (49.48 - 60.42)	40.09 (37.27 - 42.91)
	4th	25	3.76 ± 4.39	0.83 ± 1.58	55.33 ± 15.6	39.92 ± 6.41	3.75 (1.97 - 5.52)	0.89 (0.20 - 1.59)	54.76 (50.30 - 59.22)	39.39 (36.67 - 42.11)
	5th	24	3.04 ± 3.37	0.43 ± 0.90	55.96 ± 17.44	38.38 ± 5.26	3.11 (1.67 - 4.54)	0.42 (0.02 - 0.83)	55.89 (50.67 - 61.10)	38.05 (35.39 - 40.71)
	6th	21	3.90 ± 4.85	0.55 ± 1.15	55.20 ± 19.01	37.41 ± 5.88	4.41 (2.54 - 6.27)	0.62 (0.12 - 1.13)	55.94 (51.07 - 60.82)	37.65 (34.79 - 40.51)
P-trend			.07	.002	.03[k]	.29[k]	.20	.02	.05[k]	.02[k]
≥4	1st	22	5.80 ± 4.35	1.47 ± 1.61	59.26 ± 14.04	40.65 ± 7.36	5.65 (3.91 - 7.4)	1.30 (0.53 - 2.07)	60.02 (54.37 - 65.67)	39.55 (36.01 - 43.09)
	2nd	22	5.91 ± 5.13	1.43 ± 2.06	57.19 ± 13.96	41.07 ± 7.38	5.88 (3.88 - 7.88)	1.46 (0.56 - 2.35)	56.84 (51.65 - 62.04)	40.73 (37.09 - 44.36)

3rd	21	4.48 ± 4.83	1.00 ± 1.72	55.95 ± 15.29	40.27 ± 7.45	4.90 (2.83 - 6.98)	1.21 (0.53 - 1.90)	55.69 (49.69 - 61.69)	40.18 (37.00 - 43.36)
4th	19	3.84 ± 3.79	0.67 ± 1.19	58.83 ± 15.52	39.68 ± 5.55	4.53 (3.18 - 5.88)	0.77 (0.21 - 1.33)	58.76 (51.73 - 65.79)	39.92 (36.91 - 42.93)
5th	17	4.12 ± 4.96	0.44 ± 0.89	57.50 ± 16.40	37.91 ± 5.24	4.92 (3.22 - 6.61)	0.56 (0.23 - 0.89)	57.41 (50.62 - 64.20)	38.39 (35.87 - 40.91)
P-trend		.04	.003	.005[k]	.44[k]	.12	.03	.01[k]	.28[k]

[a] Home and Self-care Program (HELP) means receiving at least one structured education session (with remote digital support) comprising nursing and nutrition education and a physician consultation.

[b] CGM: continuous glucose monitoring.

[c] T1D: type 1 diabetes.

[d] SES: socioeconomic status

[e] Follow-up visit after the corresponding number of structured educations session(s).

[f] AGP: ambulatory glucose profile.

[g] TBR: time below range

[h] TIR: time in target range

[I] CV: coefficient of variation

[j] Adjusted for age, sex, body mass index, hemoglobin A1c, treatment type, diabetes duration, schooling, social status, and employment status.

[k] *P*-value (in t-test) for maintenance above reference (TIR 55.0%) and below reference (CV 40.0%).

Table S10. Effects of HELP[a] on CGM[b] metrics in T1D[c] patients with '% time CGM active' of ≥70%.

No. of education	f/u visit[d]	Subjects	AGP[e] (%)							
			at f/u visit				Adjustment model[i], 95% CI			
			TBR[f] (70)	TBR (54)	TIR[g]	CV[h]	TBR (<70 mg/dL)	TBR (<54 mg/dL)	TIR	CV
≥1	1st	33	5.55 ± 5.75	1.17 ± 1.74	65.25 ± 16.85	38.96 ± 8.78	5.52 (3.40 - 7.65)	1.18 (0.63 - 1.73)	65.29 (61.39 - 69.18)	38.89 (35.88 - 41.90)
	2nd	36	5.81 ± 4.62	1.39 ± 1.90	64.86 ± 15.64	39.83 ± 6.89	5.50 (3.92 - 7.08)	1.37 (0.71 - 2.03)	65.20 (61.10 - 69.30)	39.49 (36.98 - 42.01)
	3rd	31	5.10 ± 4.89	1.19 ± 1.74	59.52 ± 16.78	39.00 ± 6.93	5.15 (3.38 - 6.93)	1.18 (0.53 - 1.82)	59.73 (55.79 - 63.67)	38.77 (36.10 - 41.45)
	4th	27	4.67 ± 4.32	1.00 ± 1.41	57.78 ± 15.70	38.85 ± 6.39	6.79 (3.55 - 10.03)	1.33 (0.25 - 2.42)	55.43 (47.55 - 63.31)	36.21 (30.74 - 41.68)
	5th	27	4.11 ± 4.77	0.81 ± 1.86	59.27 ± 19.83	38.10 ± 7.00	3.83 (0.00 - 7.79)	0.91 (0.00 - 2.52)	59.32 (48.50 - 70.13)	36.37 (30.39 - 42.35)
	6th	25	3.68 ± 3.41	0.48 ± 0.92	59.12 ± 17.23	39.55 ± 7.76	3.64 (2.28 – 5.00)	0.47 (0.03 - 0.90)	58.61 (54.75 - 62.46)	39.35 (36.43 - 42.28)
	7th	23	3.78 ± 3.62	0.52 ± 1.08	56.96 ± 19.44	37.97 ± 5.78	3.60 (2.31 - 4.89)	0.40 (0.00 - 0.83)	56.07 (51.78 - 60.37)	37.57 (34.94 - 40.19)
	8th	18	3.72 ± 3.98	0.44 ± 0.86	59.33 ± 19.04	37.28 ± 5.56	3.62 (1.96 - 5.27)	0.42 (0.11 - 0.72)	58.84 (54.63 - 63.04)	37.15 (33.67 - 40.62)
P-trend			< .001	.001	.001[j]	.002[j]	.04	.003	.004[j]	.002[j]
≥2	1st	24	4.67 ± 3.40	0.92 ± 1.47	61.93 ± 18.53	38.83 ± 6.61	4.79 (3.16 - 6.42)	0.93 (0.37 - 1.49)	62.13 (56.31 - 67.96)	39.09 (35.83 - 42.35)
	2nd	26	5.38 ± 4.94	1.58 ± 2.18	55.54 ± 17.63	39.97 ± 6.68	5.50 (3.42 - 7.57)	1.61 (0.69 - 2.53)	56.11 (52.03 - 60.19)	39.74 (36.66 - 42.82)
	3rd	25	5.20 ± 4.71	1.24 ± 1.74	57.92 ± 17.99	39.15 ± 6.82	5.20 (3.46 - 6.94)	1.17 (0.53 - 1.81)	57.36 (52.70 - 62.02)	38.83 (35.84 - 41.83)
	4th	24	4.96 ± 4.88	1.00 ± 1.98	61.50 ± 19.02	40.33 ± 8.50	4.61 (0.44 - 8.78)	1.28 (0.00 - 3.05)	60.23 (49.99 - 70.46)	37.66 (30.88 - 44.45)
	5th	22	3.68 ± 3.64	0.55 ± 1.01	59.23 ± 17.35	38.38 ± 6.14	3.89 (0.99 - 6.79)	0.77 (0.00 - 1.64)	59.71 (52.35 - 67.08)	37.23 (31.90 - 42.55)
	6th	20	3.60 ± 3.55	0.45 ± 0.94	58.40 ± 20.25	37.52 ± 5.61	3.56 (2.32 - 4.80)	0.40 (0.03 - 0.76)	58.02 (53.51 - 62.52)	37.44 (34.66 - 40.22)
	7th	16	4.19 ± 3.97	0.50 ± 1.10	59.88 ± 18.98	37.14 ± 6.27	4.19 (2.11 - 6.27)	0.50 (0.00 - 1.09)	59.87 (55.27 - 64.48)	37.14 (33.00 - 41.29)
P-trend			.08	.04	.001[j]	.01[j]	.04	.06	.001[j]	.002[j]
≥3	1st	22	5.59 ± 5.33	1.68 ± 2.34	53.45 ± 17.75	40.71 ± 6.98	5.47 (3.48 - 7.46)	1.66 (0.61 - 2.70)	53.76 (50.13 - 57.39)	40.37 (37.40 - 43.35)
	2nd	22	5.45 ± 4.92	1.32 ± 1.84	54.95 ± 17.12	39.87 ± 6.91	5.33 (3.70 - 6.96)	1.25 (0.60 - 1.89)	55.00 (50.35 - 59.65)	39.47 (36.90 - 42.04)
	3rd	20	5.05 ± 5.27	1.15 ± 2.13	57.35 ± 18.04	40.03 ± 7.21	3.48 (0.00 - 8.03)	0.97 (0.00 - 2.95)	57.31 (45.08 - 69.53)	35.55 (29.71 - 41.40)
	4th	18	3.33 ± 3.60	0.50 ± 0.92	54.67 ± 15.48	38.96 ± 6.45	2.23 (0.00 - 5.88)	0.49 (0.00 - 1.57)	55.59 (45.70 - 65.49)	34.35 (28.54 - 40.16)
	5th	17	3.18 ± 3.24	0.35 ± 0.79	55.00 ± 19.99	37.76 ± 5.77	3.16 (1.81 - 4.50)	0.34 (0.00 - 0.76)	54.85 (49.70 - 60.00)	37.70 (34.59 - 40.81)
	6th	14	4.36 ± 4.22	0.57 ± 1.16	56.43 ± 17.63	38.00 ± 6.23	4.72 (2.67 - 6.77)	0.69 (0.03 - 1.35)	57.07 (51.50 - 62.64)	38.64 (34.94 - 42.33)
P-trend			.09	.01	.30[j]	.08[j]	.35	.03	.17[j]	.03[j]

[a] Home and Self-care Program (HELP) means receiving at least one structured education session (with remote digital support) comprising nursing and nutrition education and a physician consultation.
[b] CGM: continuous glucose monitoring.
[c] T1D: type 1 diabetes.
[d] Follow-up visit after the corresponding number of structured educations session(s).
[e] AGP: ambulatory glucose profile.
[f] TBR: time below range
[g] TIR: time in target range
[h] CV: coefficient of variation
[I] Adjusted for age, sex, body mass index, hemoglobin A1c, treatment type, diabetes duration, schooling, social status, and employment status.
[j] *P*-value (in t-test) for maintenance above reference (TIR 55.0%) and below reference (CV 40.0%).

Figure S1. Residual effects of HELP[a] on glycemic control in patients with T1D[b] after

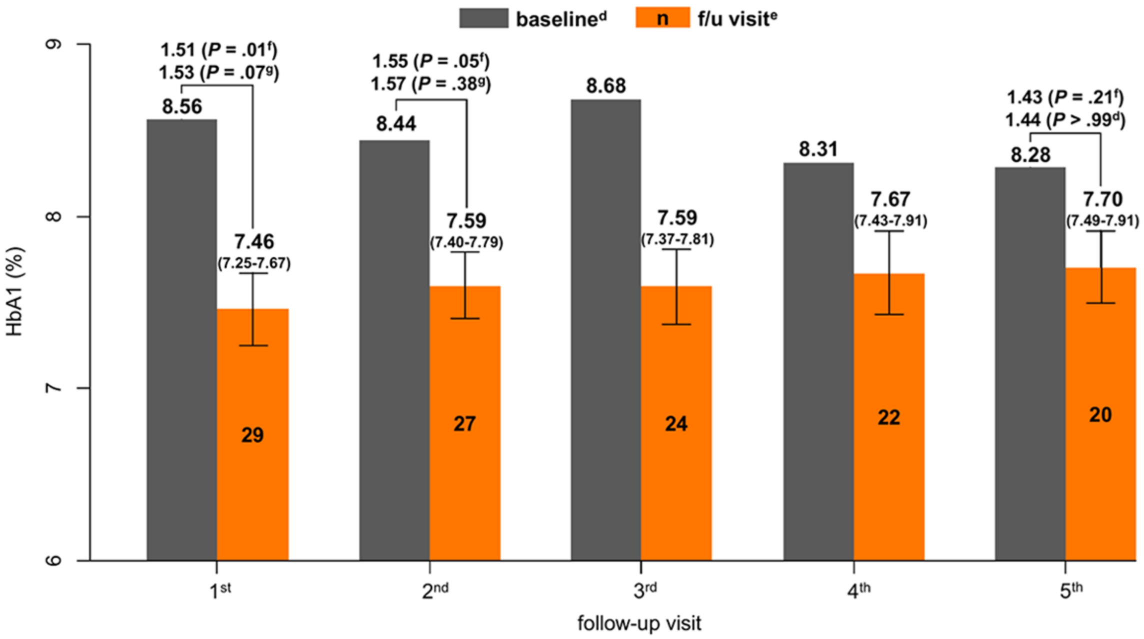

cessation of education.

[a] Home and Self-care Program (HELP) means receiving at least one structured education (with remote digital support), which includes nursing and nutrition education as well as physician consultation.

[b] T1D: type 1 diabetes.

[c]HbA1c: hemoglobin A1c.

[d] Mean values at baseline.

[e] Follow-up visit values adjusted for age, sex, body mass index, HbA1c, treatment type, and diabetes duration (with 95% confidence interval).

[f] HbA1c difference before adjustment.

[g] HbA1c difference after adjustment.

Figure S2. Long term effects of HELP[a] on CGM[b] metrics in patients with T1D[c]. TIR[d] (A), TBR[e] <70 mg/dL (B), TBR <54 mg/dL (C), and CV[f] (D)

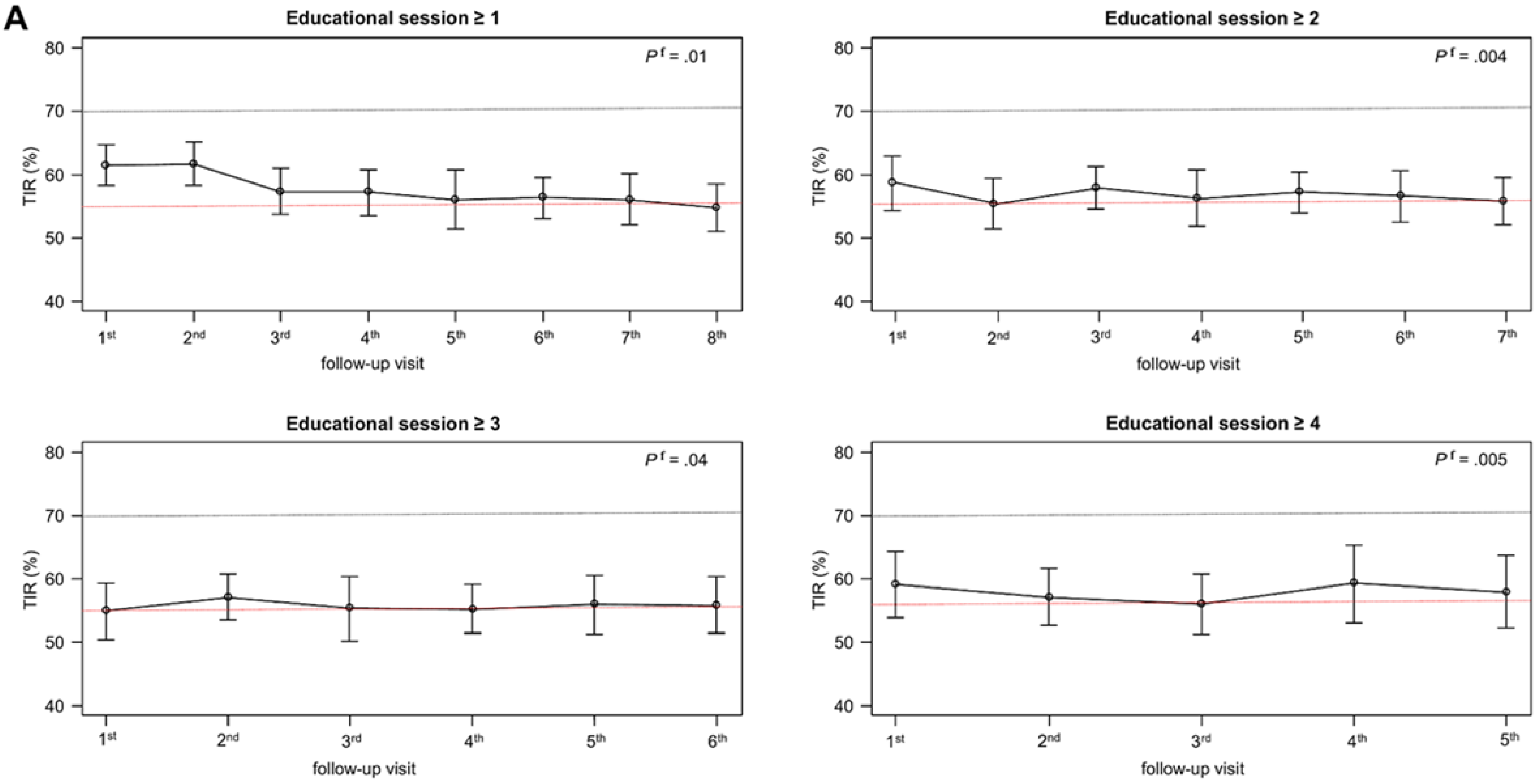

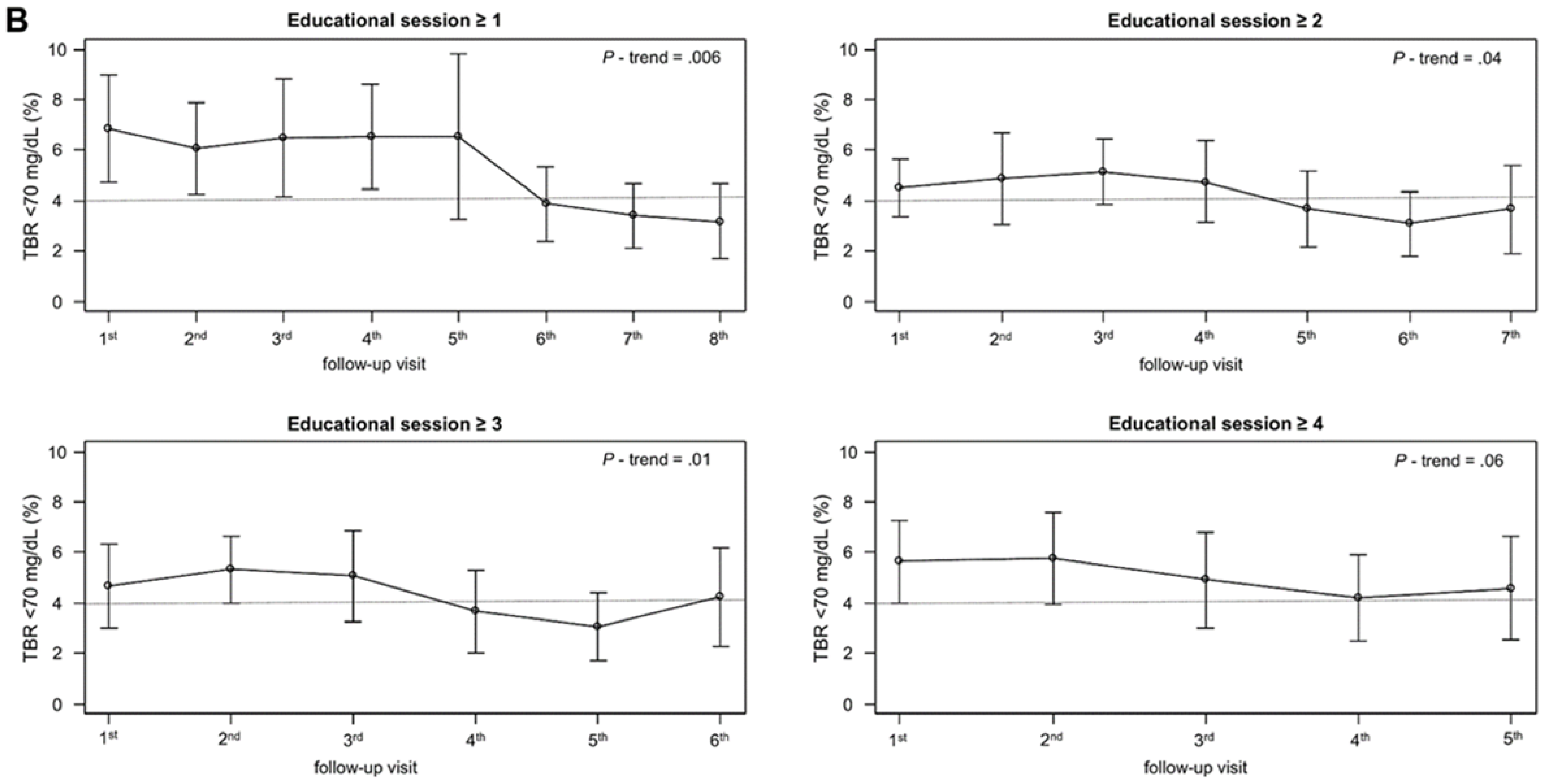
B
Educational session ≥ 1
P - trend = .006
TBR <70 mg/dL (%)
follow-up visit
Educational session ≥ 2
P - trend = .04
Educational session ≥ 3
P - trend = .01
Educational session ≥ 4
P - trend = .06

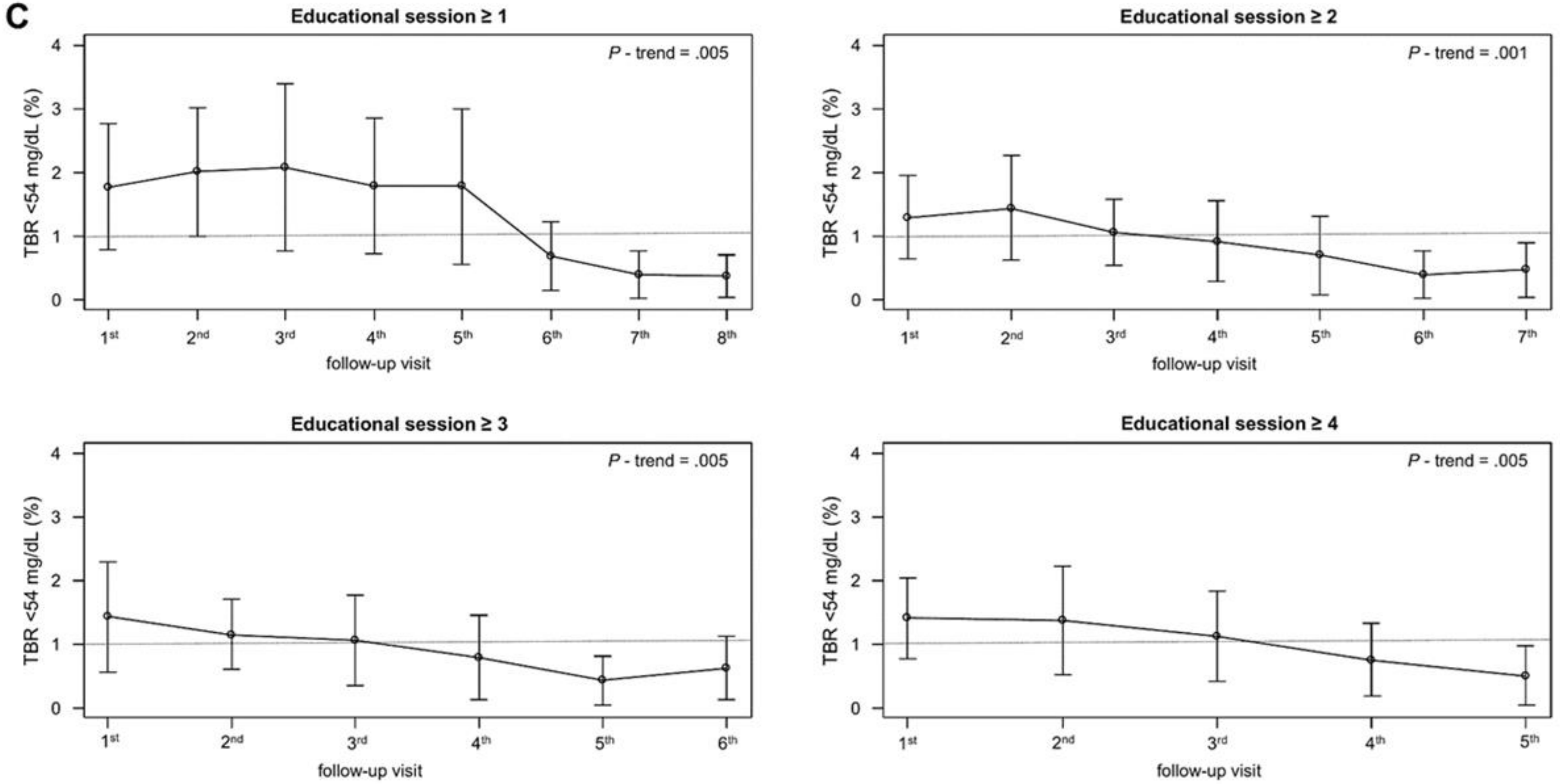

C
Educational session ≥ 1
P - trend = .005
Educational session ≥ 2
P - trend = .001
Educational session ≥ 3
P - trend = .005
Educational session ≥ 4
P - trend = .005
TBR <54 mg/dL (%)
follow-up visit

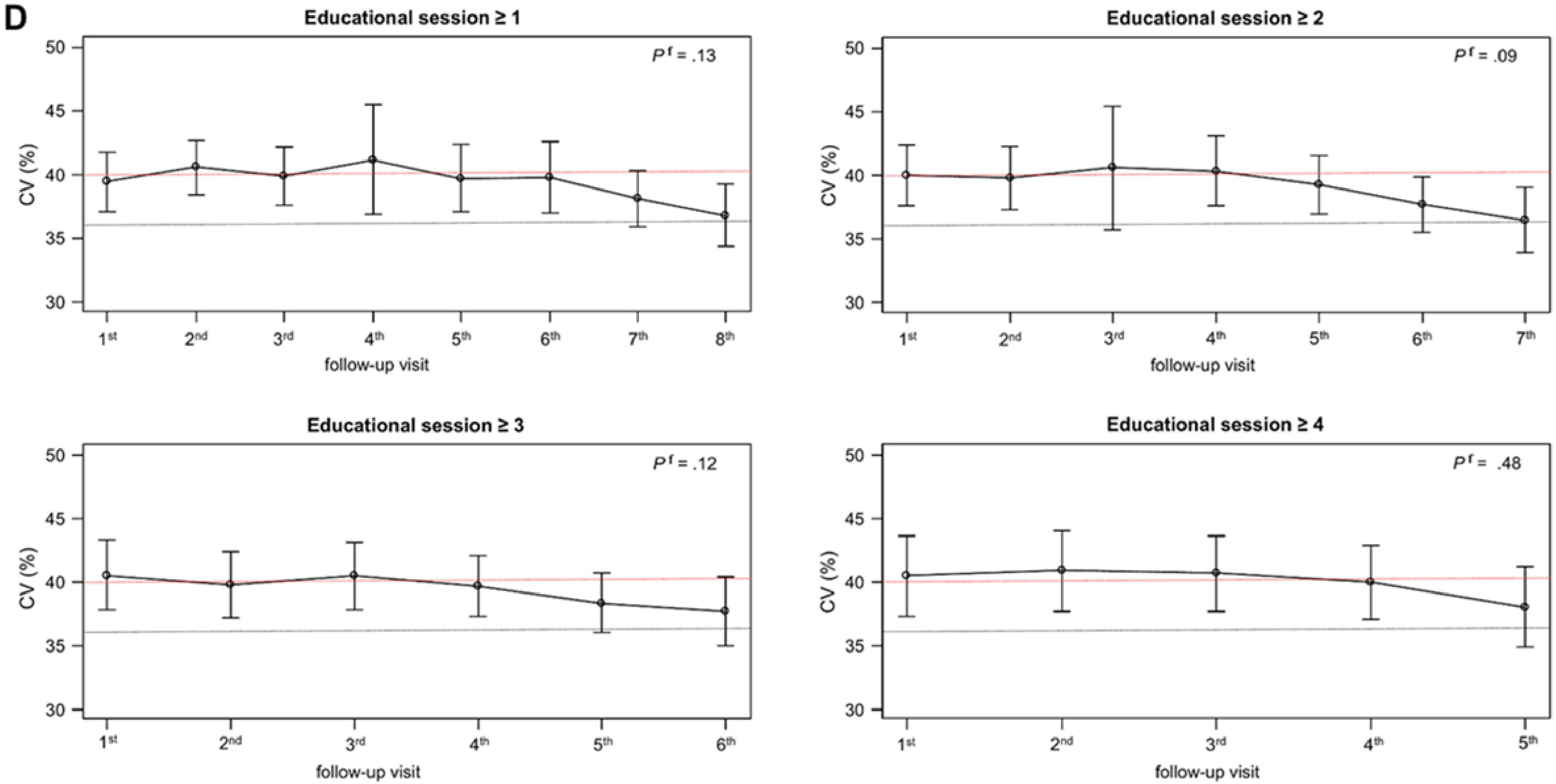

All values at follow-up visit are adjusted for age, sex, body mass index, hemoglobin A1c, treatment type, and diabetes duration. and expressed with 95% confidence interval.

[a] Home and sELf-care Program (HELP) means receiving at least one structured education (with remote digital support), which includes nursing and nutrition education as well as physician consultation.

[b] CGM: continuous glucose monitoring

[c] T1D, type 1 diabetes
[d] TIR, time in target range
[e] TBR, time below range
[f] CV, coefficient of variation
[g] *P*-value (in t-test) for maintenance above reference (TIR 55.0%) and below reference (CV 40.0%).
