# Supplementary material for: Long-Term Glycemic Control Improvement After the Home and Self-Care Program for Patients With Type 1 Diabetes: Real-World–Based Cohort Study
Source: J Med Internet Res. 2024 Sep 11;26:e60023. doi: 10.2196/60023 (PMC11425018; doi:10.2196/60023)

Long-term Glycemic Improvement After Home and Self-care Program (HELP) for Patients with Type 1 Diabetes : A Real-World based Cohort Study

'HELP' means receiving at least one in-person structured education session that includes nursing and nutrition education, accompanied by physician consultation. This program also incorporates remote support.

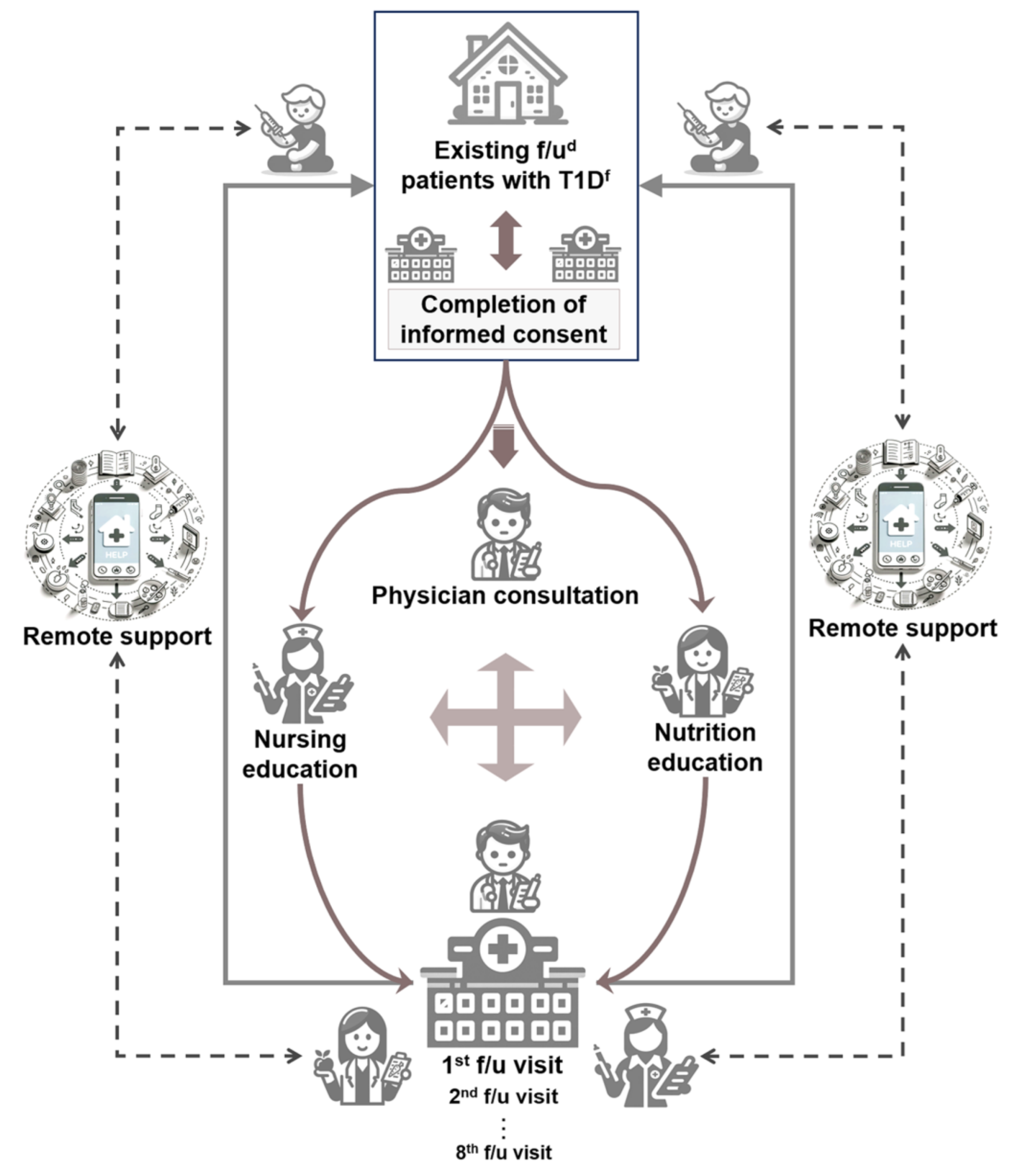

Physician consultation: up to 6 times/year
- ✓ Compliance with educations
- ✓ Empathy and emotional support
- ✓ Appraisal support (encouragement)
- ✓ Laboratory results
- ✓ AGP[b] results & CGM[c] utilization
- ✓ Hypoglycemia events
- ✓ Control of hyperglycemia
- ✓ Other complications
- ✓ Insulin dose setting
- ✓ Confirmation of lifestyle & SES[e]
- ✓ Lifestyle modifications
- ✓ Confirmation of f/u schedule

Nursing education: up to 4 times/year
- ✓ Compliance with educations
- ✓ Empathy and emotional support
- ✓ Appraisal support (encouragement)
- ✓ CGM utilization
- ✓ How to deal with hypoglycemia
- ✓ Carbohydrate counting
- ✓ Changes in lifestyle & SES
- ✓ Prevention of hypoglycemia
- ✓ Insulin dose adjustment
- ✓ How to use insulin pumps and pens
- ✓ Appropriate physical activity
- ✓ Cognitive behavioral management

Nutrition education: up to 4 times/year
- ✓ Compliance with educations
- ✓ Empathy and emotional support
- ✓ Appraisal support (encouragement)
- ✓ How to deal with hypoglycemia
- ✓ Carbohydrate counting
- ✓ Changes in lifestyle & SES
- ✓ Dietary recommendations & changes
- ✓ Appropriate food choices
- ✓ Provision of dietary table

Remote support: up to 12 times/year
- ✓ Compliance with educations
- ✓ Hypoglycemia events
- ✓ How to deal with hypoglycemia
- ✓ Control of hyperglycemia
- ✓ Carbohydrate counting
- ✓ Insulin dose adjustment
- ✓ CGM utilization
- ✓ How to use insulin pumps and pens
- ✓ Appropriate physical activity
- ✓ Appropriate food choices
- ✓ Adjustment of f/u schedule

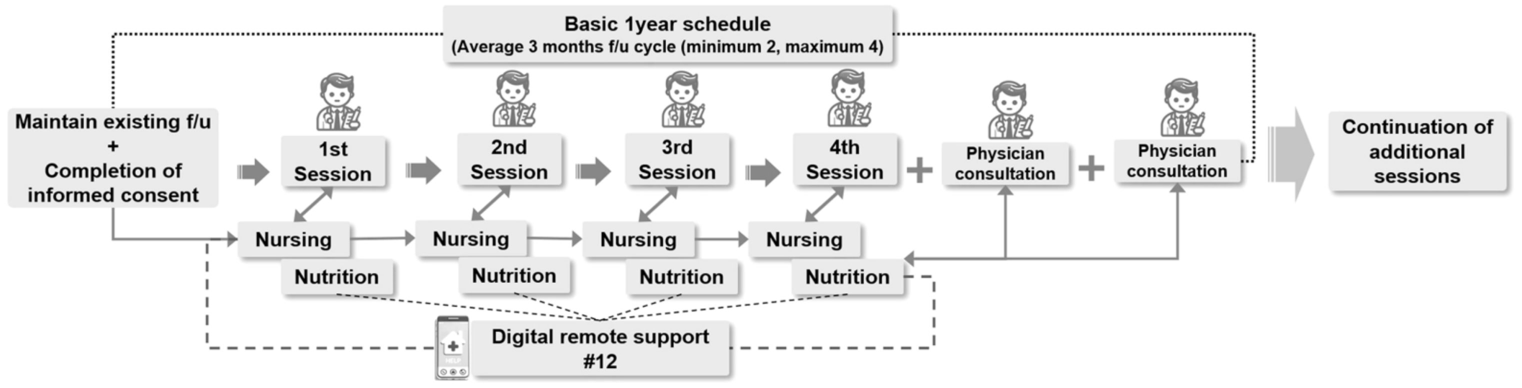

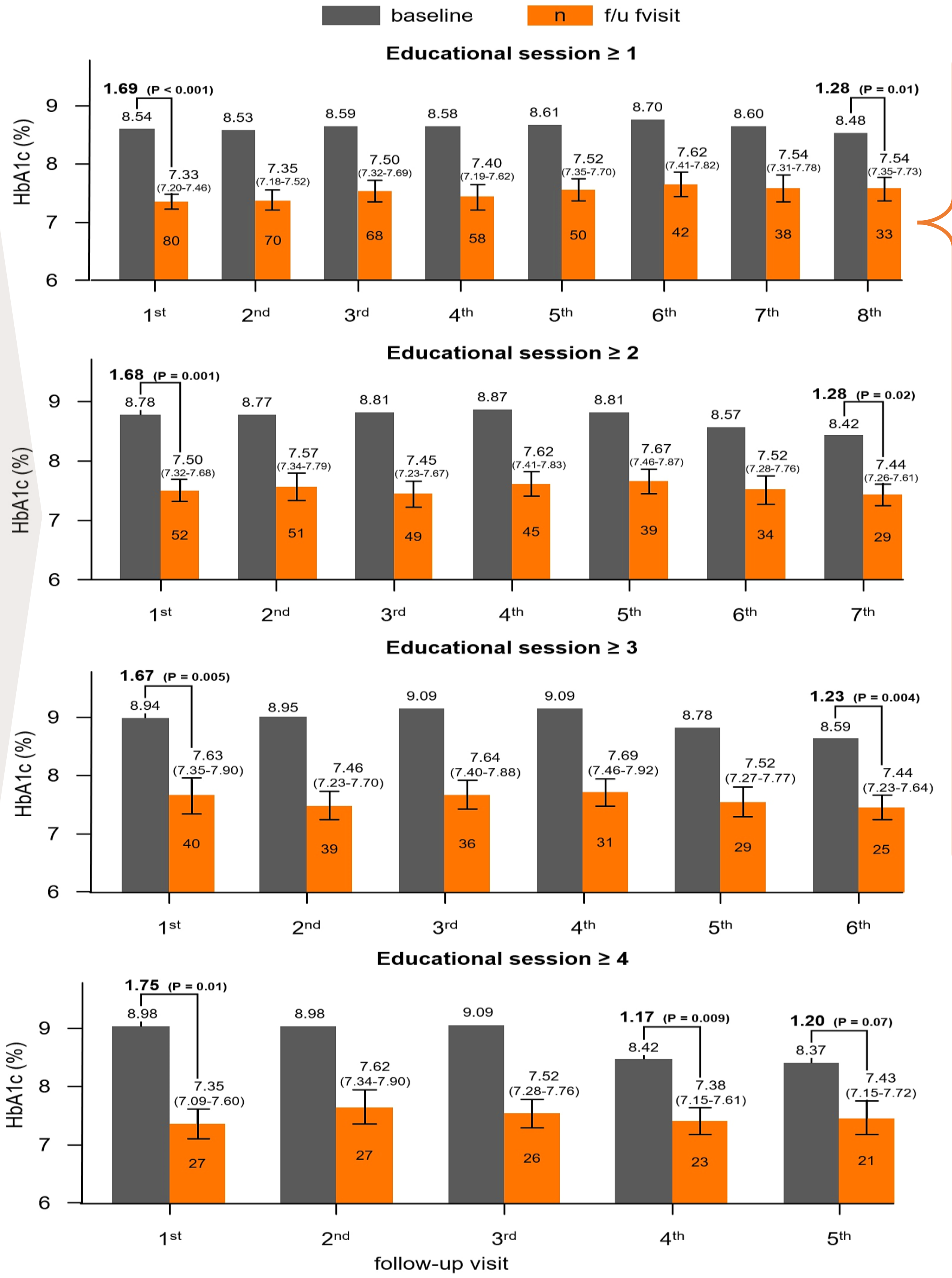

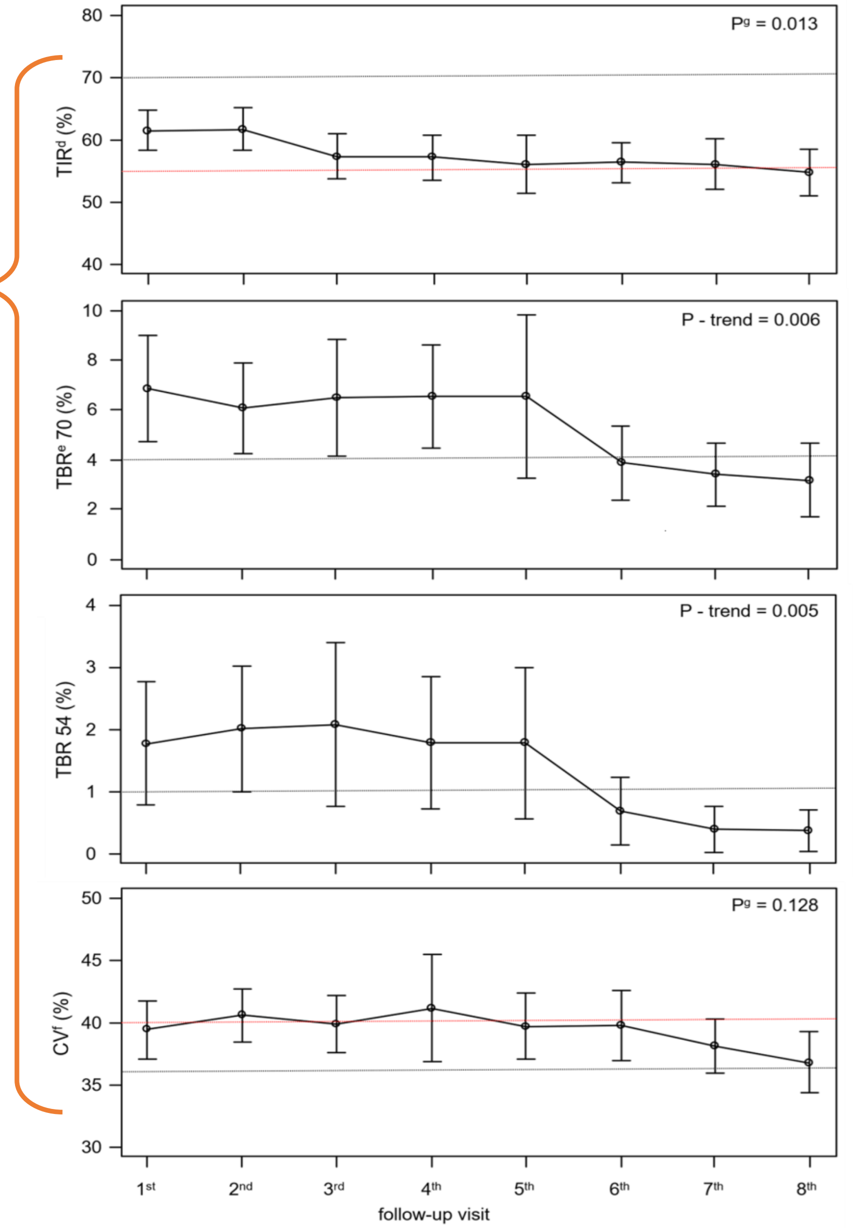

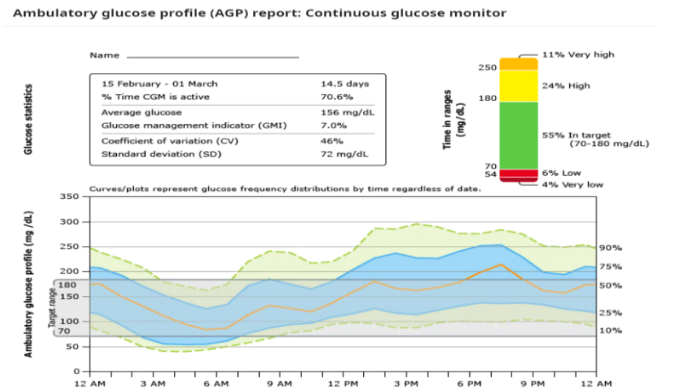

Supplement: Multimedia Appendix 4 [file jmir_v26i1e60023_app4.pdf]
